# Identification and Cluster Analysis of *Streptococcus pyogenes* by MALDI-TOF Mass Spectrometry

**DOI:** 10.1371/journal.pone.0047152

**Published:** 2012-11-07

**Authors:** Jie Wang, Na Zhou, Bin Xu, Huaijie Hao, Lin Kang, Yuling Zheng, Yongqiang Jiang, Hua Jiang

**Affiliations:** 1 State Key Laboratory of Pathogen and Biosecurity, Academy of Military Medical Sciences, Beijing, China; 2 National Center of Biomedical Analysis, Beijing, China; 3 Department of Pharmacy, Hospital of Stomatology, Jilin University, Changchun, China; The George Washington University, United States of America

## Abstract

**Background:**

Whole-cell matrix–assisted laser desorption ionization time-of-flight (MALDI-TOF) mass spectrometry (MS) has been successfully applied for bacterial identification and typing of many pathogens. The fast and reliable qualities of MALDI-TOF MS make it suitable for clinical diagnostics. MALDI-TOF MS for the identification and cluster analysis of Streptococcus pyogenes, however, has not been reported. The goal of our study was to evaluate this approach for the rapid identification and typing of S. pyogenes.

**Methods:**

65 S. pyogenes isolates were obtained from the hospital. The samples were prepared and MALDI-TOF MS measurements were conducted as previously reported. Identification of unknown spectra was performed via a pattern recognition algorithm with a reference spectra and a dendrogram was constructed using the statistical toolbox in Matlab 7.1 integrated in the MALDI Biotyper 2.0 software.

**Results:**

For identification, 61 of 65 S. pyogenes isolates could be identified correctly by MALDI-TOF MS with BioType 2.0 when compared to biochemical identification (API Strep), with an accuracy of 93.85%. In clustering analysis, 44 of 65 isolates were in accordance with those established by M typing, with a matching rate of 67.69%. When only the M type prevalence in China was considered, 41 of 45 isolates were in agreement with M typing, with a matching rate of 91.1%.

**Conclusions:**

It was here shown that MALDI-TOF MS with Soft Biotype 2.0 and its database could facilitate rapid identification of *S. pyogenes*. It may present an attractive alternative to traditional biochemical methods of identification. However, for classification, more isolates and advances in the MALDI-TOF MS technology are needed to improve accuracy.

## Introduction

Streptococcus pyogenes (GAS) is an important gram-positive pathogen. It can cause a broad spectrum of diseases, including acute pharyngitis, impetigo, rheumatic fever, bacteremia and toxic shock syndrome [1]. Timely characterization of GAS is necessary for the prevention and control of the disease, but conventional methods based on serology are often low in specificity, have a high failure rate, are time and labor consuming and are sometimes costly [2]. Therefore, a rapid and simple diagnostic tool is of utmost importance for GAS identification.

For epidemiologic study and surveillance, the M protein, a cell-surface protein that is the major virulence and immunological determinant of GAS, is the most widely used for GAS typin [3]. Effective GAS vaccine research is now focusing on the M protei [3] and it has been reported that strains of certain M types are epidemiologically associated with particular clinical syndrome [4]. Rapid and accurate discrimination of the M type is also essential for appropriate therapy and timely intervention with GAS infection.

Recently, several studies have shown that matrix–assisted laser desorption ionization time-of-flight (MALDI-TOF) mass spectrometry (MS) is a fast and reliable technique for the classification and identification of microorganism [5], [6], [7]. MALDI-TOF MS is used to examine the profile of proteins detected directly from an intact bacterial cel [8], [9]. This approach yields a reproducible spectrum within minutes. Sample preparation and analyses are easier and more time-saving and result in faster acquisition than biochemical method [6]. Here we evaluated the ability of the MALDI Biotyper 2.0 system in the identification and cluster analysis of GAS.

## Results

### Species identification

**Figure 1:**
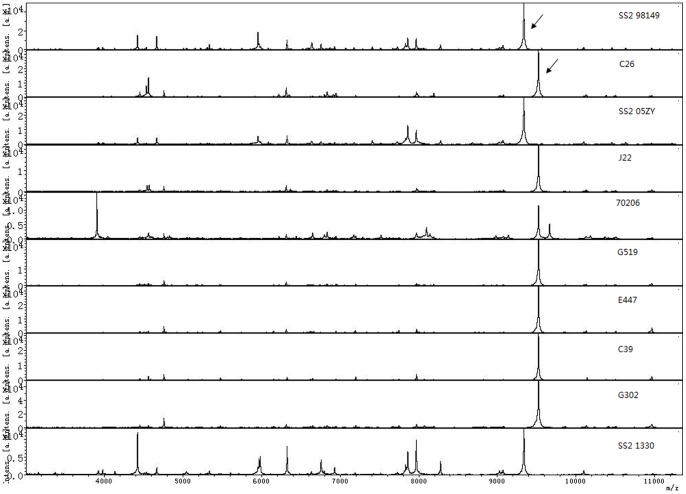
The spectra obtained from 7 GAS and 3 SS2 isolates. The peak lists of three SS2 isolates (SS2 98149, SS2 05ZY and SS2 1330) and seven GAS isolates (C26, J22, 70206, G519, E447, C39 and G302). The peaks at m/z 9378.27 and m/z 9789.30 were special to SS2 and GAS, respectively. All the isolates were confirmed by genome sequence or specific gene PCR.

In order to test the applicability and correct rate of the MALDI-TOF MS technique for GAS identification, 7 GAS isolates and 3 Streptococcus suis type 2 (SS2) isolates were first tested single blinded. The 7 GAS isolates were correctly differentiated from 3 SS2 isolates, and one conserved species characteristic peak was observed (Figure 1). Subsequently, a total of 65 GAS clinical isolates were tested, which were identified correctly at a species level, except for 94A140, J22, 70206 and 8601 (Table 1). Repeat experiments showed similar results. All four wrongly-identified isolates were re-appraised by PCR to confirm as GAS. In the correctly identified isolates, the scores for 8613 were ≤2 (1.994). Except for isolate 70206, all the isolates, including those wrongly identified (94A140, J22 and 8601), showed a similar spectra. (Fig. S1, S2).

**Table 1:** The four isolates wrongly identified by MALDI-TOF MS.

Analyte Name	Organism (best match)	Score Value	Organism (second best match)	Score Value
94A140	Neisseria polysaccharea	2.027	Neisseria flavescens	1.959
J22	streptococcus dysgalactiae	1.572	Streptococcus pyogenes	1.554
70206	staphylococcus hominis	1.987	Staphylococcus hominis ssp novobiosepticus	1.729
8601	streptococcus dysgalactiae	2.181	Streptococcus dysgalactiae ssp equisimilis	2.152

Isolates wrongly identified by MALDI-TOF MS with software Biotype 2 and its database. The meaning of Score Values was as follows: the score range between 2.3 and 3.0 means highly probable species identification; the score range between 2.0 and 2.299 means secure genus identification, probable species identification; the score range between 1.7 and 1.999 means probable genus identification; and the score range between 0 and 1.699 means no reliable identification.

### Clustering of strains

**Figure 2:**
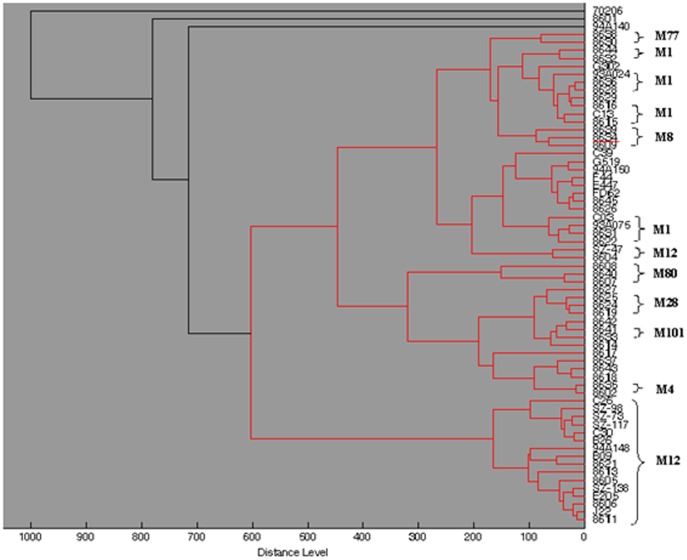
Score Oriented Dendrogram for 65 streptococcus pyogenes. Using a critical distance level of 600, the wrongly identified isolates were clustered outside of the other isolates. At a critical distance level of about 450, most isolates (16 of 18) of M12 were branched. At a critical distance level of about 270, most isolates (12 of 13) of M1 were branched, although not in one cluster group. At a critical distance level of about 200, M80, M28, M101 and M4 were grouped well.

A score oriented dendrogram was generated on the bases of the GAS reference strains in the MALDI Biotyper 2.0 database. The dendrogram revealed that clustering of 44 of 65 isolates were in accordance with the M type (Figure 2). The accuracy reached 67.69%. The cluster groups matching with the M type were M4, M28, M80, M77 and M101 with the default critical distance level of 600. Isolates of M12 were gathered within two cluster groups. The wrongly identified isolates 70206, 8601 and 94A140 were clustered outside compared with the other isolates, as expected. Surprisingly, the clustering of J22 matched its M typing.

**Figure 3:**
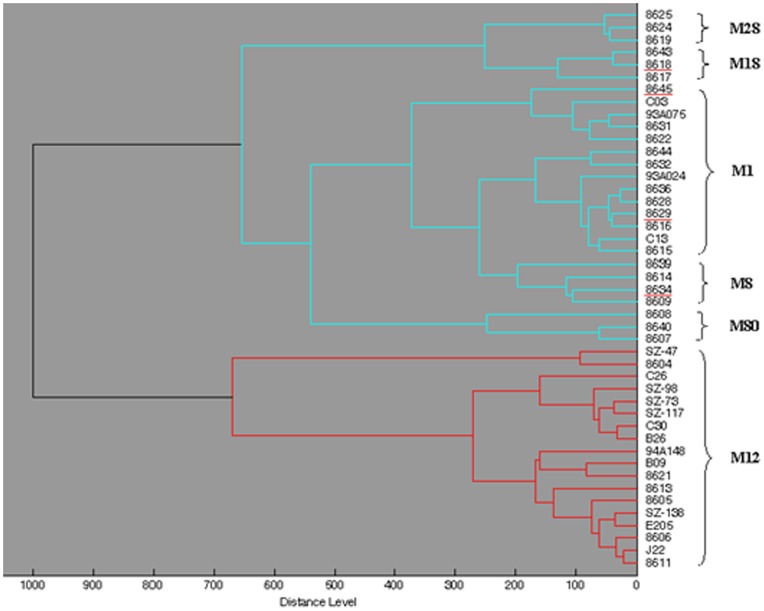
Score Oriented Dendrogram for 44 GAS with M type prevalence in China. At a critical distance level of about 670, the isolates of M12 were clustered into one group and branched compared with the other M types. However, at a critical distance level of about 280, SZ–47 and 8604 were branched, which were wrongly clustered, as shown in Figure 3. At a critical distance level of about 270, 6 groups were generated with minor error, which were M28, M18, two M1 groups, M8 and M80.

Our previous research showed that M1, M12, M8, M18, M80 and M28 were the prevalent M types (with a frequency >3% in the population) in Chin [4]. When only the M type prevalence in China was considered, only 4 isolates were not clustered in line with their M type and the accuracy reached 91.1% (Figure 3). The isolates 8629 and 8634 (M18) were clustered in M1 and M8, respectively. The isolate 8618 (M1) was clustered in the M18 group and 8645 (M8) was clustered in the M1 group. With the default critical distance level of about 660, the isolates of M12 were clearly differentiated from other M types.

## Discussion

The present study was performed to assess MALDI Biotyper 2.0 for rapid detection and cluster analysis of GAS. Initially, the influence of different cultivation conditions, such as different culture media and different growth states, were evaluated. The results showed that little influence occurred with spectra generation (data not shown). A Todd-Hewitt broth (THB) agar plate and growth overnight were used as growth conditions for follow-up tests. For sample preparation, three protocols were recommended by the MALDI Biotyper system: (i) The “direct transfer” procedure for non-pathogenic microorganisms; (ii) The “ethanol/formic acid extraction” procedure for inactivating biological material without spore formation; and (iii) The “80% TFA extraction” procedure for inactivating biological material with spore formation. Here we choose the “ethanol/formic acid extraction” procedure for GAS sample preparation. When the spectra were generated, the raw spectra were loaded into the MALDI Biotyper2.0. Then the identification and cluster analyses were conducted automatically with the default settings.

**Figure 4:**
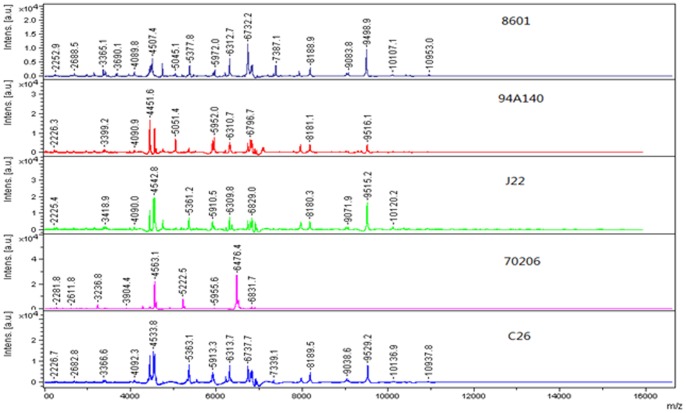
Peptide spectrum of wrongly identified GAS isolates. Isolates 8601, 94A140, J22, and 70206 were wrongly identified by MALDI-TOF MS. Isolate C26 was the positive control. No significant difference was observed between 8601, 94A140, J22 and C26. However, the peaks presented in the spectra of 70206 were obviously different from others.

Compared with biochemical methods (API strep), the results showed that MALDI-TOF MS could differentiate GAS at the species level without any complementary tests. Focusing on the peptidic profile of wrongly identified isolates, we found that, except for those of 70206, the peaks of 94A140, J22 and 8601 were similar to that of the positive control C26 (Figure 4). The peaks for 70206 were less obvious. Taking into account the fact that 70206 was evolutionarily separated earlier, we conjectured that there might be a large difference between 70206 and other strains and corresponding spectral peaks might be absent in the BioTyper 2.0 Database. For improving the accuracy of identification, multiple GAS reference spectra should be added to the database to cover the natural diversity of the species.

**Table 2:** 65 GAS isolates used in the study.

M type	code	M type	code
M1	8615	M12	B26
M1	8616	M12	C26
M1	8618	M12	C30
M1	8622	M12	E205
M1	8628	M12	J22
M1	8631	M12	SZ–47
M1	8632	M12	SZ–73
M1	8636	M12	SZ–98
M1	8644	M12	SZ–117
M1	93A024	M12	SZ–138
M1	93A075	M18	8617
M1	C03	M18	8629
M1	C13	M18	8634
M2	F44	M18	8643
M3	8601	M28	8619
M3	94A150	M28	8624
M3	94A140	M28	8625
M3	FD62	M60	70206
M4	8602	M60	E447
M4	8635	M58	G519
M6	8637	M63	G302
M8	8609	M64	8626
M8	8614	M75	8627
M8	8639	M77	8630
M8	8645	M77	8638
M12	8604	M80	8607
M12	8605	M80	8608
M12	8606	M80	8640
M12	8611	M86	8642
M12	8613	M95	C39
M12	8621	M101	8633
M12	94A148	M101	8641
M12	B09	**total**	**65 isolates**

19 M types and 65 clinical isolates were used in this paper. For M2, M6, M58, M63, M64, M75, M86 and M95, only one clinical isolate was involved. The M types in bold were those prevalent in China.

**Figure 5:**
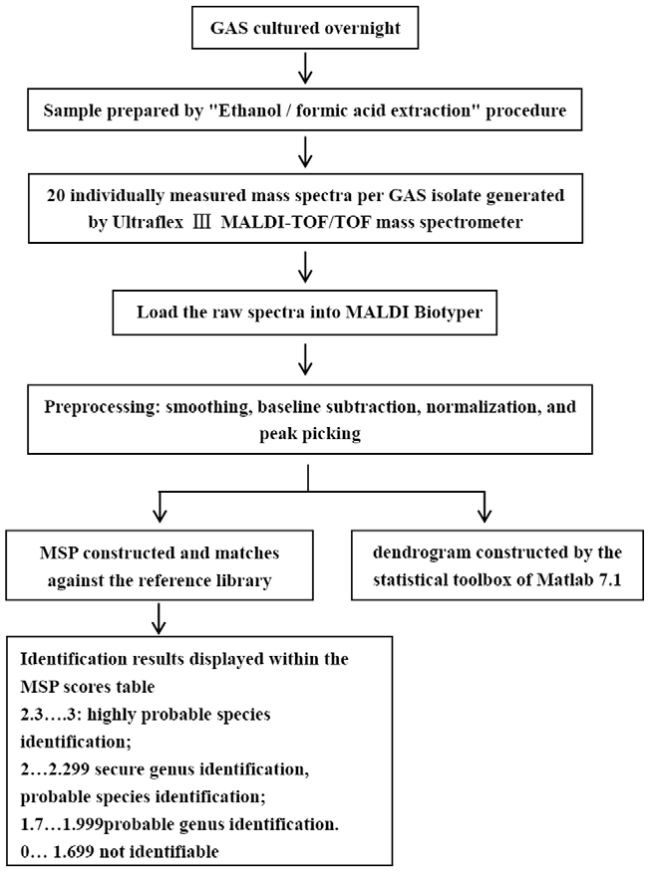
Schematic overview of the procedure involved in identification and cluster analysis.

For GAS identification, both the MALDI Biotyper system and biochemical methods (API Strep) require the prerequisite of culturing overnight. Yet, excluding the time for bacterial culturing, only 1 h was necessary for the MALDI Biotyper to perform the entire identification procedure, starting from the ethanol-formic acid extraction and ending in analysis by MALDI-TOF MS. For biochemical methods (API Strep), the results are read at 4 h for the first time. If no results are obtained, the results are read again at 24 h. In addition, for streptococcus, before API strep identification, initial assessment, such as Gram staining and a catalase test, are necessary to confirm at the genus level. Therefore, for high-throughput routine analysis, such as applications in clinical microbiology, the MALDI Biotyper system showed promising potential.

For GAS typing, the M type is the most widely used. GAS isolates are then further identified at a serotype level. The 65 GAS isolates were all within one species, and the differences were few. Differentiation of such closely related taxa are one of the constraints for MALDI-TOF MS. The reproducibility of MALDI-TOF MS is attributable to the measurement of constantly expressed, highly abundant proteins, such as ribosomal proteins, DNA-binding proteins, and cold-shock protein [10]. In this way, identification of similar samples was not difficult. However, this was not true of clustering analysis. Even so, the cluster groups still closely matched for M1, M12, M18, M28, and M80. M12 in particular was always well differentiated (Figures 2 and 3). For other M types, a much higher standardization of cultivation and more refined methods of sample preparation and measurement are needed to generate high-quality spectra set [11]. This would enhance the match rate. More clinical isolates are also needed, especially for M types, which were only found to contain 1–3 isolates in this paper. For the M types that are prevalent in China, MALDI-TOF MS can be used to differentiate the M types of isolates at a match rate of up to 91.1%. More clinical isolates are needed for verification.

In addition to the above factors associated with MS, the features of M proteins might also affect the cluster analysis of GAS. First, the highly variable N terminal of the M protein was the molecular basis for the GAS M type. Extensive allelic variation within this region has been reported for many M serotypes, such as M3 [12], M1 [13] and M28 [14]. The alterations within the coding region might lead to a change in amino acid residues, resulting in a change of the m/z (mass-to-charge ratio) of the protein detected, which might result in different cluster groups for the same M type. Secondly, the emm-type covered only 100 amino acids of the N terminal region of the mature M protein. Regarding the differences in other regions of the M protein, such as the C repeat region [15], [16], the variable number would also lead to a change in the m/z and lead to an emm type mismatch. Thirdly, the proteins identified by MALDI-MS were not only housekeeping proteins but included many other types of protein [10]. Regarding GAS, it was reported that prophages accounted for the vast majority of gene content differences between GAS strains [14]. Except for the extracellular virulence factors, the prophage also encoded DNases, a phospholipase A2, macrolide resistance and a surface protein [14]. Therefore, with different prophages, the protein detected might be different, and might result in a mismatch of M types.

Although MALDI-TOF MS performed well for GAS identification, this method also has certain limitations, such as the misidentification of some pathogens with the absence of corresponding spectra in the database, differentiation of very closely related taxa (such as GAS), and analyses of mixed samples. These limitations would be overcome by database expansion and technological progress in the future. However, the instrumentation is costly. The reagent cost per test is very low and there are no consumable costs. Of course, knowledge in MS is essential for applying the procedure. To summarize, future applications of MALDI-TOF MS in clinical microbiology is encouraging.

In summary, we used 65 GAS isolates in this study to assess the MALDI Biotyper 2.0 for identification and relationship analysis based on spectra generated with Ultraflex mass spectrometers. Our results confirm that the MALDI Biotyper system for identification is rapid and accurate. Although misidentification occurred when compared with biochemical methods, further expansion of the database with a larger number of clinical isolates will be very important for future use in clinics. Clustering analysis was suited well in this research for the determination of M type prevalence in China. For clustering of other M types, except for testing on a large collection of GAS of diverse origins, refining the methods of sample preparation to improve the quality of mass spectra might also be important.

## Materials and Methods

### Ethics statement

All the experiments in the paper were conducted under the supervision of the ***Institutional Review Board of the Academy of Military Medical Sciences.*** All the bacterial isolates were kindly provided by the hospital, Institutes and Academy. No samples were collected from patients directly and therefore the study was exempt from obtaining informed consent. All relevant ethical safeguards have been met in the experiments.

### Bacterial isolates

A total of 65 GAS isolates from patients with GAS infections were collected from Beijing Children's Hospital, Peking Union Medical College Hospital, Beijing Community Hospital, the National Institute for the Control of Pharmaceutical and Biological Products, the Chinese Centre for Disease Control and Prevention, the PLA 304^th^ Hospital and the Academy of Military Medical Sciences.

The clinical symptoms involved glomerulonephritis, scarlet fever, suppurative tonsillitis, pleurisy and other unknown factors. Strains that have been isolated were mainly from specimens of blood (1 isolate), epipharynx (33 isolates), pus (3 isolates), wounds (1 isolate), urine (2 isolates), sputum (9 isolates), vaginal secretions (5 isolates) and other sources (11 isolates). All strains were grown on THB plates in 5% CO_2_ and at 37°C overnight. All isolates were identified with API streps, specific gene PCR analysis and the M type was confirmed by emm-gene sequencing (Table 2).

### Sample preparation and MS identification

Sample preparation for MALDI-TOF MS was performed as previously describe [6]. After overnight growth on the THB agar plates, the clones were harvested and resuspended in 300 μL MilliQ purified water. Then 900 μL ethanol was added to inactivate the pathogens. The suspension was collected by centrifugation at 13,000 rpm for 2–5 min. If necessary, the pellet was centrifuged another time to remove the ethanol completely. Then 50 μL of 70% formic acid was added and thoroughly mixed to disrupt the cell walls. Subsequently, 50 μL acetonitrile was added for protein extraction. Then the mixture was centrifuged at 13,000 rpm for 2 min. 2 μL of supernatant were transferred to the sample slide and were dried at room temperature. Then, the sample was overlaid with 2 μL matrix (a-cyano-4-hydroxycinnamic acid in 50% acetonitrile/2.5% trifluoroacetic acid) and dried again.

Measurements were performed on an Ultraflex III MALDI-TOF/TOF mass spectrometer (Bruker Daltonics, Leipzig, Germany) equipped with a 337 nm nitrogen laser. Spectra were recorded in the linear positive mode at a laser frequency of 200 Hz within a mass range of 2000 Da to 20,000 Da. The IS1 voltage was 25 kV and the IS2 voltage was maintained at 23.5 kV. The lens voltage was 6.5 kV and the pulse ion extraction time was 100 ns. To achieve an acceptable average, a measurement of about 20 spectra per sample was necessary.

### Data analysis

The main spectra (MSPs) were the fundamental components on which spectrum identification in the MALDI Biotyper was based. When the raw spectra were loaded into the soft Biotyper2, they were preprocessed to construct MSPs that included five steps: mass adjustment, smoothing, baseline subtraction, normalization and peak picking. The methods chosen for each step were as follows: 1) The mass adjustment step applied Spectra Compressing by a factor of 10; 2) The smoothing step applied the Savitsky-Golay algorithm with a frame size of 25 Da to the mass adjusted data; 3) The baseline correction step applied the Minimum for finding the baseline; 4) The normalization step applied the Maximum Norm to normalize the baseline subtracted data; and 5) The peak picking step applied a spectra differentiation algorithm for finding the peaks. In addition, during the generation of MSPs, a recalibration of spectra was necessary if mass shifts of the peaks occurred. For identification, the unknown MSPs were compared with reference spectra stored in the Biotyper 2.0 database based on a pattern recognition algorithm using peak positions, peak intensity distributions and peak frequencies.

In taxonomy trees, MSPs are assigned to hierarchically ordered taxon nodes. The hierarchical clustering uses a dendrogram-based algorithm to form tree-like structures from the distance of the species and links them together with a linkage algorithm. Depending on the selection of the respective distance and linkage algorithm, different dendrograms can be generated. Two different methods are available to form clusters: i) prediction of the cluster number, and ii) selection of a cut-off level based on distances. Here we used K Means' algorithms. The dendrogram was constructed using the statistical toolbox of Matlab 7.1 (MathWorks Inc., USA) integrated in the MALDI Biotyper 2.0 software.

### Criteria for Identification of Isolates

The identification results, in terms of matching MSPs and the corresponding final logarithmic score values, are displayed within the MSP Scores Table. The Meanings of Score Values were as follows: log (score) values ≥2.3 and <3 were required for highly probable species identification and values between <2.299 and ≥2 for secure genus identification and probable species identification. The above produced good results. Results based on log (score) values of <1.999 and ≥1.7 meant probable genus identification. And the scores <1.7 were rated as not identifiable by the software. The procedure involved in identification and cluster analysis were shown in figure 5.

## Supporting Information

Figure S1 **Alignment of raw spectra for M1, M12, M8, M18, M80 and M28.** Intensity of ions is shown on the y axis and the m/z of each peak is indicated on the x axis with range 2,000 to 20,000. The m/z values of the peaks were assigned manually. (A), (B) MALDI-TOF mass spectra for thirteen M1 type isolates. (C), (D) MALDI-TOF mass spectra for eighteen M12 type isolates. (E) MALDI-TOF mass spectra for four M8 type isolates. (F) MALDI-TOF mass spectra for four M18 type isolates. (G) MALDI-TOF mass spectra for three M80 type isolates. (H) MALDI-TOF mass spectra for three M28 type isolates.
(TIF)

Figure S2 **Alignment of raw spectra for M2, M3, M4, M6, M60, M58, M63, M64, M75, M77, M86, M95, M101.** Intensity of ions is shown on the y axis and the m/z of each peak is indicated on the x axis with range 2,000 to 20,000. The m/z values of the peaks were assigned manually. (A) MALDI-TOF mass spectra for one M2 type isolate. (B) MALDI-TOF mass spectra for four M3 type isolates. (C) MALDI-TOF mass spectra for two M4 type isolates. (D) MALDI-TOF mass spectra for one M6 type isolate. (E) MALDI-TOF mass spectra for two M60 type isolates. (F) MALDI-TOF mass spectra for one M58 type isolate. (G) MALDI-TOF mass spectra for one M63 type isolate. (H) MALDI-TOF mass spectra for one M64 type isolate. (I) MALDI-TOF mass spectra for one M75 type isolate. (J) MALDI-TOF mass spectra for two M77 type isolates. (K) MALDI-TOF mass spectra for one M86 type isolate. (L) MALDI-TOF mass spectra for one M95 type isolate. (M) MALDI-TOF mass spectra for two M101 type isolates.
(TIF)

Table S1 **Peaklist for M1 type isolates (part1).** m/z – intensity values of top 50 major peaks were listed. It included six isolates of M1 type (8615, 8616, 8618, 8622, 8628, 8631).
(DOCX)

Table S2 **Peaklist for M1 type isolates (part2).** m/z – intensity values of top 50 major peaks were listed. It includes six isolates of M1 type (8632, 8636, 8644, 93A024, 93A075, C03).
(DOCX)

Table S3 **Peaklist for M1, M2 and M3 type isolates.** m/z – intensity values of top 50 major peaks were listed. It includes one M1 type isolate (C13), one M2 type isolate (F44) and four M3 type isolates (8601, 94A150, 94A140, FD62).
(DOCX)

Table S4 **Peaklist for M4, M8 type isolates.** m/z – intensity values of top 50 major peaks were listed. It includes two M4 type isolates (8602,8635) and four M8 type isolates (8609, 8614, 8639, 8645).
(DOCX)

Table S5 **Peaklist for M12 type isolates (part 1).** m/z – intensity values of top 50 major peaks were listed. It includes six isolates of M12 type (8604, 8605, 8606, 8611, 8613, 8621).
(DOCX)

Table S6 **Peaklist for M12 type isolates (part 2).** m/z – intensity values of top 50 major peaks were listed. It includes six isolates of M12 type (94A148, B09, B26, C26, C30, E205).
(DOCX)

Table S7 **Peaklist for M12 type isolates ((part 3).** m/z – intensity values of top 50 major peaks were listed. It includes six isolates of M12 type (J22, SZ–47, SZ–73, SZ–98, SZ–117, SZ–138).
(DOCX)

Table S8 **Peaklist for M18 and M60 type isolates.** m/z – intensity values of top 50 major peaks were listed. It includes four M18 type isolates (8617, 8629, 8634, 8643) and two M60 isolates (70206, E447).
(DOCX)

Table S9 **Peaklist for M28 and M80 type isolates.** m/z – intensity values of top 50 major peaks were listed. It includes three M28 type isolates (8619, 8624, 8625) and three M80 type isolates (8607, 8608, 8640).
(DOCX)

Table S10 **Peaklist for M77, M101, M6 and M58 type isolates.** m/z – intensity values of top 50 major peaks were listed. It includes two M77 type isolates (8630, 8638), two M101 type isolates (8633, 8641), one M6 type isolate (8637) and one M58 type isolate (G519).
(DOCX)

Table S11 **Peaklist for M63, M64, M75, M86 and M95 type isolates.** m/z – intensity values of top 50 major peaks were listed. It includes one M63 type isolates (G302), one M64 type isolates (8626), one M75 type isolates (8627), one M86 type isolates (8642), one M95 type isolates (C39).
(DOCX)
