# Supplementary figures and images for: Identification and Cluster Analysis of Streptococcus pyogenes by MALDI-TOF Mass Spectrometry
Source: PLoS One. 2012 Nov 7;7(11):e47152. doi: 10.1371/journal.pone.0047152 (PMC3492366; doi:10.1371/journal.pone.0047152)

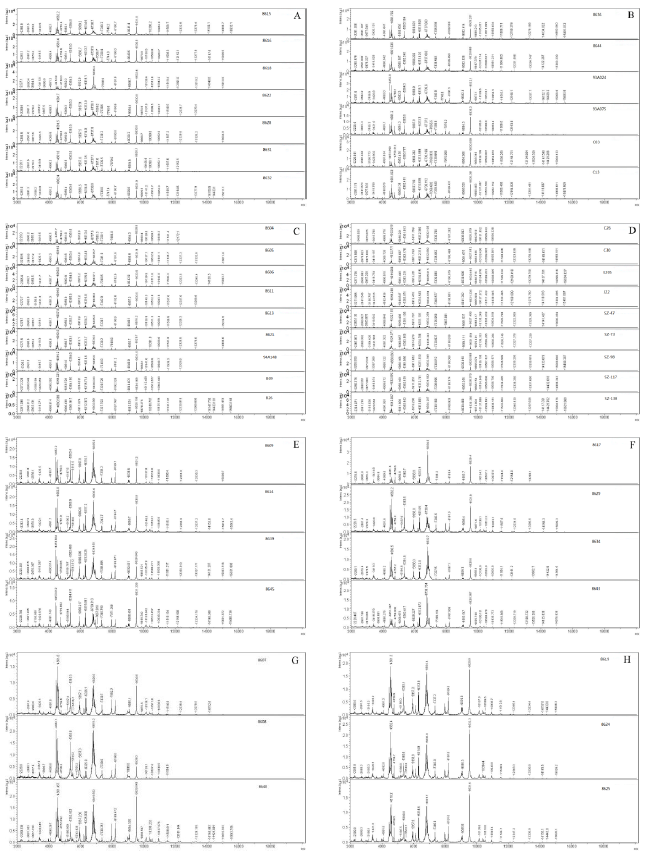

Supplement: Figure S1 — Alignment of raw spectra for M1, M12, M8, M18, M80 and M28. Intensity of ions is shown on the y axis and the m/z of each peak is indicated on the x axis with range 2,000 to 20,000. The m/z values of the peaks were assigned manually. (A), (B) MALDI-TOF mass spectra for thirteen M1 type isolates. (C), (D) MALDI-TOF mass spectra for eighteen M12 type isolates. (E) MALDI-TOF mass spectra for four M8 type isolates. (F) MALDI-TOF mass spectra for four M18 type isolates. (G) MALDI-TOF mass spectra for three M80 type isolates. (H) MALDI-TOF mass spectra for three M28 type isolates. (TIF) [file pone.0047152.s001.tif]

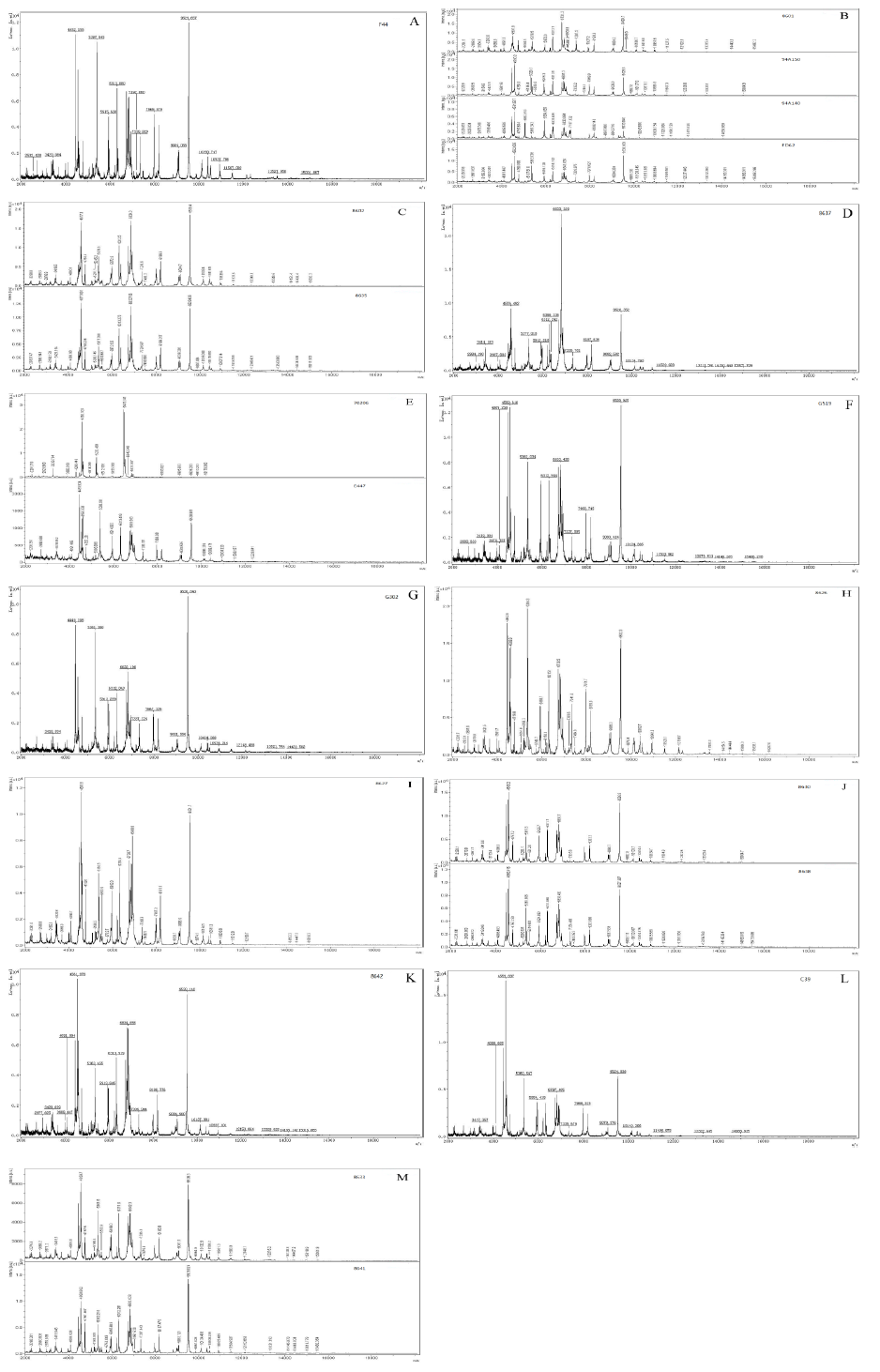

Supplement: Figure S2 — Alignment of raw spectra for M2, M3, M4, M6, M60, M58, M63, M64, M75, M77, M86, M95, M101. Intensity of ions is shown on the y axis and the m/z of each peak is indicated on the x axis with range 2,000 to 20,000. The m/z values of the peaks were assigned manually. (A) MALDI-TOF mass spectra for one M2 type isolate. (B) MALDI-TOF mass spectra for four M3 type isolates. (C) MALDI-TOF mass spectra for two M4 type isolates. (D) MALDI-TOF mass spectra for one M6 type isolate. (E) MALDI-TOF mass spectra for two M60 type isolates. (F) MALDI-TOF mass spectra for one M58 type isolate. (G) MALDI-TOF mass spectra for one M63 type isolate. (H) MALDI-TOF mass spectra for one M64 type isolate. (I) MALDI-TOF mass spectra for one M75 type isolate. (J) MALDI-TOF mass spectra for two M77 type isolates. (K) MALDI-TOF mass spectra for one M86 type isolate. (L) MALDI-TOF mass spectra for one M95 type isolate. (M) MALDI-TOF mass spectra for two M101 type isolates. (TIF) [file pone.0047152.s002.tif]
