# Supplementary material for: Identification and Cluster Analysis of Streptococcus pyogenes by MALDI-TOF Mass Spectrometry
Source: PLoS One. 2012 Nov 7;7(11):e47152. doi: 10.1371/journal.pone.0047152 (PMC3492366; doi:10.1371/journal.pone.0047152)
Supplement: Table S1 — Peaklist for M1 type isolates (part1). m/z – intensity values of top 50 major peaks were listed. It included six isolates of M1 type (8615, 8616, 8618, 8622, 8628, 8631). (DOCX) [file pone.0047152.s003.docx]

Table S1. Peaklist for M1 type isolates (part1).

|  | 8615 | | 8616 | | 8618 | | 8622 | | 8628 | | 8631 | |
| --- | --- | --- | --- | --- | --- | --- | --- | --- | --- | --- | --- | --- |
| No | m/z | Intens. | m/z | Intens. | m/z | Intens. | m/z | Intens. | m/z | Intens. | m/z | Intens. |
| 1 | 4562.2 | 24950.12 | 4561.4 | 16780.96 | 6836.4 | 29491.79 | 4561.7 | 13840.43 | 4561.5 | 19879.48 | 9530.1 | 15702.14 |
| 2 | 9531 | 15559.77 | 9530.5 | 15458.09 | 9532.4 | 21344.14 | 9530.8 | 12768.15 | 4452 | 15281.69 | 5363 | 11980.8 |
| 3 | 4452.7 | 12945.04 | 4451.7 | 11175.4 | 6739.3 | 9228.23 | 4452.1 | 9470.39 | 5363 | 13642.26 | 4561.6 | 11854.66 |
| 4 | 4590.3 | 10032.52 | 5363 | 10046.95 | 6929.9 | 8529.34 | 5363.2 | 7359.35 | 9530 | 12268.85 | 4452 | 11465 |
| 5 | 5364 | 9717.48 | 4589.5 | 7614.98 | 6315.1 | 8283.52 | 6313.9 | 6076.47 | 6737.9 | 9449.77 | 6737.1 | 7032.34 |
| 6 | 6314.5 | 8533.67 | 6313.7 | 7268.44 | 4760 | 7146.23 | 6738.1 | 5600.17 | 6844.5 | 8554.61 | 6313.5 | 6507.51 |
| 7 | 6738.7 | 8478.44 | 6737.9 | 7045.51 | 4452.8 | 6820.19 | 6845 | 5035 | 6801.6 | 8545.94 | 6801.3 | 6248.26 |
| 8 | 6845.3 | 8290.71 | 6845 | 6524.56 | 4578.2 | 6547.69 | 4758.7 | 5022.68 | 6314 | 7470.72 | 6845.1 | 5981.86 |
| 9 | 6802.2 | 7306.29 | 6801.4 | 6058.99 | 4562.5 | 6210.88 | 6801.8 | 4911.84 | 4589.3 | 7444.13 | 4758.9 | 4841.06 |
| 10 | 5958.2 | 6525.4 | 5956.7 | 5369.98 | 5364.3 | 5589.35 | 4589 | 4515.22 | 5957.5 | 6486.77 | 7970 | 4398.91 |
| 11 | 6900.5 | 5779.61 | 6899.9 | 4502.47 | 8191.9 | 5501.13 | 5957.9 | 4185.7 | 6898.7 | 4944.77 | 5957 | 4373.74 |
| 12 | 8190.7 | 5078.38 | 4758.9 | 4258.88 | 5379.7 | 5378.98 | 6899.9 | 3056.18 | 8190 | 4786.95 | 7339.5 | 3943.6 |
| 13 | 4759.4 | 4434.64 | 8190 | 4099.88 | 3415.8 | 3732.94 | 8190.6 | 2876.82 | 6946.1 | 4452.49 | 4589.6 | 3810.74 |
| 14 | 7340.2 | 4183.06 | 6945.6 | 3364.9 | 7988.2 | 3409.69 | 6946.5 | 2679.99 | 4758.6 | 3590.09 | 8190.3 | 3650.29 |
| 15 | 6947.6 | 4054.34 | 7338.4 | 3189.96 | 5932.6 | 3319.15 | 7970.6 | 2506.6 | 5914.3 | 3372.8 | 6946.4 | 3203.91 |
| 16 | 7970.8 | 3918.6 | 7970.3 | 3186.18 | 6221.8 | 3302.62 | 7339.6 | 2098.87 | 7969.8 | 3361.59 | 5913.8 | 2333.57 |
| 17 | 4514.8 | 3689.86 | 6219.6 | 2489.11 | 5959 | 2950.55 | 5915.5 | 1866.48 | 7338.2 | 3335.57 | 6900 | 2193.14 |
| 18 | 5916.2 | 3103.85 | 5914.4 | 2357.55 | 4516 | 2779.06 | 6220.1 | 1858.67 | 3420.5 | 2412.39 | 3419.9 | 1936.06 |
| 19 | 6221.3 | 2998.18 | 4515.3 | 2138.92 | 5916.6 | 2715.2 | 3367.2 | 1784.56 | 6220.3 | 2276.88 | 4514.6 | 1910.17 |
| 20 | 9041.4 | 2288.17 | 5320.8 | 1871.13 | 4537.1 | 2512.24 | 3419.3 | 1776.08 | 3366.7 | 2031.93 | 9084.6 | 1740.29 |
| 21 | 9084.1 | 2084.98 | 9040.6 | 1765.5 | 5973.7 | 2198.39 | 2681.7 | 1309.17 | 9039.2 | 1934.13 | 3366.7 | 1569.37 |
| 22 | 3420.5 | 2045.38 | 9084.3 | 1694.95 | 3367.8 | 2173.4 | 3397.8 | 1298.58 | 2681 | 1869.64 | 2681.6 | 1554.09 |
| 23 | 5318.3 | 1957.41 | 3419.5 | 1600.3 | 9086.7 | 2101.67 | 4090.7 | 1263.72 | 9084.4 | 1728.54 | 4090.3 | 1464.57 |
| 24 | 4092.1 | 1779.47 | 2681.2 | 1486.17 | 7398.9 | 2026.61 | 9084.4 | 1230.32 | 3398.7 | 1609.84 | 6220.1 | 1437.23 |
| 25 | 3367.9 | 1756.49 | 3366.9 | 1330.61 | 9041.4 | 1893.05 | 9040.7 | 1196.35 | 4089.9 | 1591.18 | 9037.9 | 1411.26 |
| 26 | 5186.8 | 1531.81 | 4089.4 | 1187.02 | 4091.3 | 1829.93 | 2280.5 | 1177.3 | 5465.8 | 1534.27 | 5465.9 | 1342.91 |
| 27 | 10390.2 | 1507.89 | 5186.2 | 1131.66 | 5516.9 | 1669.17 | 3667.5 | 1073.58 | 2280.9 | 1455.2 | 3667.1 | 1276.86 |
| 28 | 2681.5 | 1482.64 | 3398.8 | 1122.78 | 3463.5 | 1537.83 | 5186.1 | 1030.66 | 2226.1 | 1306.01 | 5186.6 | 1252.57 |
| 29 | 5246.9 | 1467.8 | 6350.3 | 1117.22 | 10139.4 | 1352.48 | 2226.4 | 986.36 | 5185.6 | 1164.95 | 3398.2 | 1231.57 |
| 30 | 10137 | 1418.69 | 10138.5 | 1111.6 | 5248 | 1287.44 | 3155.5 | 938.59 | 3980.9 | 1145.92 | 3981.5 | 1225.03 |
| 31 | 3399.1 | 1343.2 | 5515 | 1093.51 | 5316.7 | 1219.55 | 3472 | 927.72 | 3665.4 | 1123.6 | 10390.1 | 1147.17 |
| 32 | 3666.9 | 1314.33 | 2281.1 | 1042.77 | 5062.2 | 1219.49 | 3981 | 895.02 | 3471 | 1071.1 | 2027 | 1136.57 |
| 33 | 2280.9 | 1306.7 | 2225.3 | 1037.02 | 2690.4 | 1152.71 | 5466.1 | 893.1 | 2977.6 | 1067.23 | 2226.1 | 1091.06 |
| 34 | 5515.6 | 1276.54 | 3666.6 | 1022.72 | 3989.5 | 1107.44 | 2978.2 | 878.91 | 5059.9 | 880.48 | 10138.2 | 1048.46 |
| 35 | 5467.7 | 1197.56 | 5466.4 | 995.3 | 5186.6 | 1064.34 | 5060.3 | 849.9 | 10390 | 857.58 | 8830.6 | 1021.6 |
| 36 | 3982 | 1188.54 | 10390.7 | 985.5 | 3155.9 | 1063.18 | 5245.7 | 822.19 | 10135.4 | 830.43 | 2280.6 | 1013.57 |
| 37 | 5062.1 | 1177.68 | 5245.5 | 964.54 | 5460.3 | 1044.55 | 10137 | 723.14 | 2377 | 791.64 | 5060.5 | 997.89 |
| 38 | 2978.6 | 956.5 | 3979.6 | 908.54 | 10392 | 1037.69 | 10390.1 | 655.42 | 3154.5 | 787.66 | 10952.1 | 968.14 |
| 39 | 10954 | 927.92 | 10105.6 | 841.29 | 9636.9 | 952.59 | 5741.8 | 524.98 | 10952 | 777.93 | 5246.3 | 944.11 |
| 40 | 10511.9 | 924.98 | 5061.1 | 838.11 | 3696.9 | 947.71 | 10509.5 | 454.31 | 3641.3 | 709.5 | 6651.3 | 923.43 |

Table S1. Cont.

|  | 8615 | | 8616 | | 8618 | | 8622 | | 8628 | | 8631 | |
| --- | --- | --- | --- | --- | --- | --- | --- | --- | --- | --- | --- | --- |
| No | m/z | Intens. | m/z | Intens. | m/z | Intens. | m/z | Intens. | m/z | Intens. | m/z | Intens. |
| 41 | 3155.3 | 912.67 | 3156.2 | 817.96 | 2227.1 | 789.81 | 7056.2 | 440.57 | 2530.8 | 691.27 | 3472.2 | 910.8 |
| 42 | 2227.2 | 905.01 | 8830.5 | 815.52 | 10512.9 | 763.8 | 10953.7 | 416.26 | 10507.7 | 668.55 | 10106.6 | 908.31 |
| 43 | 8832.5 | 608.35 | 2978.2 | 807.52 | 3172.3 | 738.96 | 9634.8 | 397.04 | 7480.9 | 425.76 | 2978.3 | 873.69 |
| 44 | 7201 | 585.42 | 10953.7 | 804.69 | 2964.8 | 661.27 | 7482.1 | 390.66 | 9868.7 | 322.46 | 3154.8 | 831.63 |
| 45 | 7483.2 | 540.07 | 3447.5 | 785.56 | 10942.2 | 642.97 | 8831 | 307.02 | 11507.2 | 297.35 | 7747.7 | 787.43 |
| 46 | 5747.9 | 461.17 | 3468.6 | 746.83 | 5750.5 | 591.73 | 7748.6 | 302.2 | 12328.6 | 151.02 | 2532.9 | 779.89 |
| 47 | 7746.2 | 431.6 | 2532.1 | 680.85 | 3108.9 | 532.67 | 8067.7 | 292.14 | 12155.3 | 146.54 | 9634.7 | 761.63 |
| 48 | 11508.7 | 413.93 | 9633.2 | 665.99 | 7494.8 | 517.84 | 9870.6 | 248.63 | 13328.2 | 95.8 | 5743.6 | 717.2 |
| 49 | 12332.9 | 179.03 | 5742.1 | 655.11 | 11519.4 | 153.48 | 11510.5 | 179.84 | 13276.5 | 86.93 | 7199 | 682.69 |
| 50 | 12154.6 | 165.1 | 10508.8 | 562.07 | 12161 | 127.39 | 12331.7 | 97.19 | 13530.8 | 66.35 | 6622.5 | 666.17 |

m/z - intensity values of top 50 major peaks were listed. It includes six isolates of M1 type (8615, 8616, 8618, 8622, 8628, 8631).
