# Supplementary material for: Identification and Cluster Analysis of Streptococcus pyogenes by MALDI-TOF Mass Spectrometry
Source: PLoS One. 2012 Nov 7;7(11):e47152. doi: 10.1371/journal.pone.0047152 (PMC3492366; doi:10.1371/journal.pone.0047152)
Supplement: Table S2 — Peaklist for M1 type isolates (part2). m/z – intensity values of top 50 major peaks were listed. It includes six isolates of M1 type (8632, 8636, 8644, 93A024, 93A075, C03). (DOCX) [file pone.0047152.s004.docx]

Table S2. Peaklist for M1 type isolates (part2).

|  | | 8632 | | 8636 | | 8644 | | | | 93A024 | | 93A075 | | | C03 | | |
| --- | --- | --- | --- | --- | --- | --- | --- | --- | --- | --- | --- | --- | --- | --- | --- | --- | --- |
| No | m/z | | Intens. | m/z | Intens. | | m/z | Intens. | m/z | | Intens. | | m/z | Intens. | | m/z | Intens. |
| 1 | 4562.4 | | 18522.72 | 4561.7 | 13663.77 | | 4561.5 | 14222.54 | 9532.1 | | 21716.78 | | 9530.3 | 13311.73 | | 9530.8 | 17021.71 |
| 2 | 9531 | | 11253.83 | 9529.2 | 10325.83 | | 9529.7 | 10005.4 | 4452.8 | | 16379.85 | | 4561.2 | 9629.76 | | 4759.3 | 9149.11 |
| 3 | 4452.7 | | 9567.84 | 4452.3 | 9582.89 | | 4451.9 | 7209.23 | 5364.3 | | 13136.63 | | 4452.2 | 8170.18 | | 4451.9 | 6864.26 |
| 4 | 4577.9 | | 7881.34 | 5363.2 | 7747.34 | | 4576.8 | 6298.46 | 6739.6 | | 11502.88 | | 5363.5 | 7033.08 | | 5363.8 | 6609.54 |
| 5 | 4590.1 | | 7199.14 | 6313.6 | 5597.8 | | 6737.1 | 5798.91 | 6315.1 | | 10233.9 | | 4759.1 | 6538.93 | | 4562.2 | 5953 |
| 6 | 6738.9 | | 6762.68 | 6737.7 | 5267.97 | | 5362.3 | 4571.34 | 6803 | | 9826.91 | | 6313.1 | 5187.99 | | 6313.7 | 3973.69 |
| 7 | 4759.5 | | 6246.68 | 4589.7 | 5067.97 | | 6313.4 | 4522.69 | 4590.7 | | 8876.75 | | 6737.3 | 4567.54 | | 6738.2 | 3822.54 |
| 8 | 6314.6 | | 5801.03 | 6843.4 | 4671.37 | | 4589.3 | 4491 | 6845 | | 8652.97 | | 4590 | 4398.38 | | 6800.8 | 3462.71 |
| 9 | 5363.9 | | 5660.34 | 6801.7 | 4654 | | 6844.3 | 4441.8 | 5959 | | 8469.47 | | 6801.5 | 4250.44 | | 6845.5 | 3256.69 |
| 10 | 6845.2 | | 5437.8 | 4577.9 | 3664.06 | | 6899.3 | 3904.17 | 8191.5 | | 7433.59 | | 6845.2 | 4150.83 | | 5958.4 | 2770.89 |
| 11 | 6801.3 | | 5120.11 | 5956.8 | 3646.34 | | 5956.2 | 3645.06 | 6901.6 | | 6420.6 | | 5957.9 | 3219.02 | | 7970.9 | 2419.6 |
| 12 | 6900.7 | | 4915.95 | 4759 | 3190.34 | | 6801.7 | 3626.91 | 7971.9 | | 5453.52 | | 7970.3 | 2985.61 | | 8190.2 | 2102.2 |
| 13 | 5958.5 | | 4707.64 | 6900.5 | 3053 | | 4758.2 | 3444.06 | 4760.1 | | 5249.71 | | 6947.1 | 2590.92 | | 6945.1 | 1880.69 |
| 14 | 8190.7 | | 3412.47 | 8189.5 | 3008.23 | | 8189.5 | 3031.94 | 6947.9 | | 4978.63 | | 8190.1 | 2573.34 | | 6220.4 | 1861.86 |
| 15 | 6947.3 | | 2860.94 | 7338.9 | 2663.6 | | 6816.4 | 2817.6 | 5916.5 | | 4378.87 | | 6899.3 | 2253.86 | | 4536.6 | 1818.43 |
| 16 | 7971 | | 2650.05 | 6946.2 | 2601.03 | | 4535.5 | 2177.83 | 6222.1 | | 3292.2 | | 7338.9 | 2181.8 | | 7339.7 | 1778.37 |
| 17 | 3420.8 | | 2509.84 | 7969.6 | 2292.63 | | 4512.7 | 2124.17 | 9042.4 | | 3207.51 | | 5915.7 | 1943.55 | | 2681.5 | 1692.23 |
| 18 | 3367.3 | | 2453.83 | 5914.2 | 2000.89 | | 5377.3 | 1878.06 | 9086.7 | | 3082.6 | | 3421 | 1937.55 | | 4590.9 | 1681.54 |
| 19 | 4091.2 | | 2052.52 | 6218.9 | 1997.4 | | 5378.9 | 1865.43 | 10139.6 | | 2334.57 | | 4479 | 1918.39 | | 2028.2 | 1665.97 |
| 20 | 5916.3 | | 2049.26 | 3420.1 | 1797.34 | | 6943.9 | 1752.51 | 7340.9 | | 2318.82 | | 3366.7 | 1757.53 | | 3420.9 | 1664.91 |
| 21 | 2281 | | 2047.7 | 4512.9 | 1792.69 | | 7985.4 | 1701.03 | 10393.6 | | 2181.79 | | 4091.1 | 1598.93 | | 3367.9 | 1476.71 |
| 22 | 6220.8 | | 2016.72 | 5320.9 | 1499.29 | | 6219.2 | 1619.46 | 3420.8 | | 2061.29 | | 2681.3 | 1575.79 | | 5915.8 | 1433.26 |
| 23 | 5187.9 | | 1843.74 | 3367.4 | 1393.71 | | 3419.6 | 1592.49 | 4092 | | 2009.44 | | 5187.4 | 1545.18 | | 4091.6 | 1401.11 |
| 24 | 3399.2 | | 1794.72 | 2681.4 | 1340.6 | | 5914 | 1397.4 | 2682.2 | | 1808.64 | | 6220.8 | 1467.49 | | 4514.4 | 1366.6 |
| 25 | 9039.6 | | 1539.85 | 9082.4 | 1272.49 | | 4090.6 | 1287.06 | 5187.9 | | 1785.44 | | 3399.5 | 1447.61 | | 3398.2 | 1352.63 |
| 26 | 9085.3 | | 1506.82 | 9039.1 | 1232.89 | | 7969.9 | 1248.83 | 4560.5 | | 1640.77 | | 9084.3 | 1397.61 | | 2226.7 | 1309.14 |
| 27 | 3156.2 | | 1463.71 | 2281.3 | 1221.37 | | 2281 | 1239.34 | 3366 | | 1528.2 | | 9040.5 | 1341.3 | | 3981 | 1259.06 |
| 28 | 3448.5 | | 1415.94 | 3667.3 | 1164.57 | | 5930.4 | 1237.11 | 3398.6 | | 1483.85 | | 3981.5 | 1303.23 | | 6900.4 | 1167.09 |
| 29 | 2681.3 | | 1407.63 | 4091.6 | 1122.69 | | 3365.6 | 1183.03 | 2281.4 | | 1394.06 | | 3667.2 | 1293.56 | | 5186.7 | 1163.54 |
| 30 | 3982 | | 1394.81 | 2226.7 | 1048.63 | | 9082.8 | 1036.86 | 10513.1 | | 1382.78 | | 2226.5 | 1236.61 | | 9084.3 | 1159.86 |
| 31 | 2977.7 | | 1352.13 | 6352.2 | 1003.63 | | 9038.6 | 1035.69 | 3981.9 | | 1347.08 | | 5466 | 1228.76 | | 5466.9 | 1064.77 |
| 32 | 2226 | | 1287.55 | 3154.8 | 922.11 | | 5187.6 | 1019.57 | 10954.5 | | 1338.6 | | 3155.3 | 1216.83 | | 3156.8 | 1062.51 |
| 33 | 7339.8 | | 1275.84 | 5185.1 | 911.83 | | 2681 | 934.94 | 5466.7 | | 1229.69 | | 8832.9 | 1173.75 | | 3172.5 | 1060.51 |
| 34 | 5247.5 | | 1255.66 | 5245.2 | 836.66 | | 3989 | 843.43 | 3156.5 | | 1197.43 | | 5245.5 | 1095.56 | | 2281.3 | 1058.31 |
| 35 | 10141.2 | | 1104.48 | 5467.6 | 821.69 | | 5246.8 | 842.66 | 5062.2 | | 1180.17 | | 5061.2 | 1087.37 | | 3668.3 | 1031.2 |
| 36 | 10390.7 | | 1087.94 | 10135.3 | 795.74 | | 2226 | 830.66 | 5514.2 | | 1178.2 | | 3470.3 | 1041.2 | | 9039.8 | 973.2 |
| 37 | 5059.4 | | 1085.66 | 10387.7 | 734.23 | | 5516.7 | 773.14 | 2226 | | 1116.08 | | 6651.4 | 1026.71 | | 5062.4 | 961.34 |
| 38 | 5466.1 | | 1052.83 | 2977 | 730.6 | | 5060.6 | 725.71 | 2977.8 | | 985.84 | | 2280.5 | 1005.19 | | 5247.4 | 946.09 |
| 39 | 3667.3 | | 867.03 | 5060.5 | 705.03 | | 3154.9 | 704.94 | 2027.3 | | 954.66 | | 5742.8 | 990.22 | | 2978.9 | 879.23 |
| 40 | 7199.9 | | 668.11 | 3981.6 | 695.03 | | 2976.2 | 694.46 | 11510.1 | | 793.5 | | 10136.9 | 946.73 | | 5045.5 | 859.4 |

Table S2. Cont.

|  | 8632 | | 8636 | | 8644 | | 93A024 | | 93A075 | | C03 | |
| --- | --- | --- | --- | --- | --- | --- | --- | --- | --- | --- | --- | --- |
| No | m/z | Intens. | m/z | Intens. | m/z | Intens. | m/z | Intens. | m/z | Intens. | m/z | Intens. |
| 41 | 7485.1 | 621.59 | 5516.7 | 543.11 | 10137.2 | 692.26 | 3641.1 | 781.23 | 10391.3 | 902.33 | 5323.1 | 849.89 |
| 42 | 2756 | 605.67 | 5741.2 | 520.26 | 5465.9 | 685.8 | 5742.2 | 744.24 | 2978.6 | 899.26 | 8832.9 | 846.94 |
| 43 | 5740.8 | 604.43 | 10950.4 | 510.43 | 10390.1 | 621.03 | 8831.4 | 678.69 | 6065.1 | 884.79 | 6341.7 | 702.23 |
| 44 | 7747.4 | 576.16 | 2752.4 | 499.03 | 7338.5 | 597.06 | 9873.1 | 674.09 | 10106.6 | 857.4 | 3109.7 | 695.03 |
| 45 | 10510.7 | 553.14 | 10511.9 | 474.63 | 3666.5 | 522.23 | 7198.7 | 612.42 | 7746.8 | 855.16 | 10138.2 | 694.09 |
| 46 | 10953.3 | 551.53 | 8829.1 | 451.63 | 5743.3 | 433.09 | 7481.2 | 590.13 | 2027.7 | 853.98 | 10108.7 | 685.37 |
| 47 | 9635.3 | 483.6 | 7198.3 | 344.63 | 2531.7 | 432.17 | 7748.8 | 543.32 | 3171.5 | 795.41 | 10390.5 | 683.11 |
| 48 | 8831.7 | 412.77 | 7484.7 | 321.66 | 10508.6 | 379.31 | 12158.1 | 392.82 | 6152.5 | 793.24 | 6064.9 | 658.14 |
| 49 | 11509.7 | 349.97 | 9869.2 | 292.03 | 7200.9 | 376.94 | 12335 | 310.58 | 10953.4 | 735.94 | 5742.3 | 597.74 |
| 50 | 9869.5 | 208.92 | 11506.7 | 223.69 | 7485.5 | 312.97 | 13337.7 | 212.48 | 7483.2 | 700.74 | 12156.7 | 581.66 |

m/z - intensity values of top 50 major peaks were listed. It includes six isolates of M1 type (8632, 8636, 8644, 93A024, 93A075, C03).

.
