# Supplementary material for: Identification and Cluster Analysis of Streptococcus pyogenes by MALDI-TOF Mass Spectrometry
Source: PLoS One. 2012 Nov 7;7(11):e47152. doi: 10.1371/journal.pone.0047152 (PMC3492366; doi:10.1371/journal.pone.0047152)
Supplement: Table S3 — Peaklist for M1, M2 and M3 type isolates. m/z – intensity values of top 50 major peaks were listed. It includes one M1 type isolate (C13), one M2 type isolate (F44) and four M3 type isolates (8601, 94A150, 94A140, FD62). (DOCX) [file pone.0047152.s005.docx]

Table S3. Peaklist for M1, M2 and M3 type isolates.

|  | | C13 | | | F44 | | 8601 | | | 94A150 | | | | 94A140 | | | FD62 | |
| --- | --- | --- | --- | --- | --- | --- | --- | --- | --- | --- | --- | --- | --- | --- | --- | --- | --- | --- |
| No | m/z | | Intens. | m/z | | Intens. | | m/z | Intens. | | m/z | Intens. | m/z | | Intens. | m/z | | Intens. |
| 1 | 4561.8 | | 14866.14 | 9529.7 | | 11946.17 | | 6731.3 | 15831.64 | | 9529.6 | 9876.5 | 4451.6 | | 18941.64 | 9530.9 | | 12407.99 |
| 2 | 9530.4 | | 14826.11 | 4452.2 | | 11030.71 | | 9499.7 | 13510.05 | | 6313.6 | 8007.59 | 4561.2 | | 14574.19 | 4562 | | 12083.38 |
| 3 | 4452 | | 7788.23 | 5397.6 | | 10481.06 | | 4507.9 | 8177.87 | | 6801.3 | 7969.76 | 6796.6 | | 8753.76 | 4452.7 | | 8654.82 |
| 4 | 5362.5 | | 7311.4 | 4561.9 | | 8368.91 | | 6313.1 | 7973.51 | | 6834.1 | 7504.8 | 6829.2 | | 8575.97 | 5363.7 | | 7630.41 |
| 5 | 6313.8 | | 6540.91 | 6313.9 | | 6940.71 | | 6844 | 7527.41 | | 6738.1 | 7244.03 | 5952.1 | | 8567.62 | 6314.2 | | 5066.69 |
| 6 | 4758.8 | | 6362.71 | 6738.1 | | 6723.23 | | 6817.1 | 6240.34 | | 5915.3 | 6511.13 | 5912.1 | | 7556.67 | 6845 | | 4634.25 |
| 7 | 6738.1 | | 6286.46 | 6844.8 | | 6273.31 | | 4743.5 | 5939.39 | | 7969.8 | 6312.47 | 5051.4 | | 7533.31 | 6834.7 | | 4600.93 |
| 8 | 4589.9 | | 6120.89 | 6801.4 | | 6214.89 | | 7387.5 | 5792.13 | | 6946.5 | 4980.21 | 4473.3 | | 6320.44 | 6802 | | 4481.6 |
| 9 | 6845.1 | | 5960.14 | 6835 | | 5556.6 | | 5378.5 | 5449.31 | | 8189.8 | 4917.01 | 6733.4 | | 6277.12 | 6738.7 | | 4402.22 |
| 10 | 4577.5 | | 5720.66 | 7969.6 | | 4933.63 | | 4492.2 | 4652.7 | | 6899.7 | 4674.41 | 6311 | | 5778.59 | 4758.8 | | 3800.58 |
| 11 | 6836.1 | | 4664.49 | 5915.6 | | 4753.77 | | 4479.9 | 4637.73 | | 5957 | 4362.7 | 9516 | | 4595.4 | 7970.6 | | 3488.47 |
| 12 | 6899.8 | | 4559.66 | 8189.7 | | 4145.14 | | 8189.6 | 4581.4 | | 4590.2 | 3582.81 | 8180.9 | | 4475.68 | 6900.2 | | 3190.32 |
| 13 | 5957.7 | | 4424.29 | 6946.4 | | 3639.66 | | 4450.9 | 4232.66 | | 6220.4 | 2541.5 | 6326.9 | | 4434.56 | 6947.3 | | 2905 |
| 14 | 6800.9 | | 4309.14 | 6899.9 | | 3420.14 | | 5972.8 | 3690.81 | | 4515.4 | 2314.11 | 7962.3 | | 4316.23 | 5916.1 | | 2881.7 |
| 15 | 8189.4 | | 3680.8 | 7339.8 | | 3237.4 | | 3365.8 | 3264.45 | | 9039.9 | 2043.74 | 6893 | | 4198.57 | 5957.7 | | 2862.87 |
| 16 | 7339.6 | | 2995.8 | 4759.3 | | 2902.91 | | 4536 | 2821.3 | | 4758.9 | 1951.99 | 4597.5 | | 3567.92 | 8189.8 | | 2720.83 |
| 17 | 4537.2 | | 2842.26 | 4589.4 | | 2380.46 | | 5363.2 | 2766.14 | | 9082.7 | 1930.7 | 6940.9 | | 3490.18 | 7340 | | 2370.52 |
| 18 | 5379.1 | | 2823.43 | 5957.3 | | 2354.66 | | 7927.2 | 2547.55 | | 5320.1 | 1884.26 | 7086.1 | | 2936.6 | 4590.5 | | 2206.84 |
| 19 | 6219.7 | | 2804.89 | 9084.1 | | 2209.14 | | 5957.5 | 2487.05 | | 3420.5 | 1526.92 | 5360.3 | | 2509.8 | 2681.6 | | 1382.56 |
| 20 | 4516.3 | | 2542.37 | 9039.7 | | 1769.71 | | 3419.1 | 2355.72 | | 10137 | 1445.85 | 7110.7 | | 2326.13 | 6220.3 | | 1381.79 |
| 21 | 6946.3 | | 2487.97 | 10390.7 | | 1699.77 | | 5930.3 | 2145.05 | | 3367.2 | 1415.16 | 4090.4 | | 1751.68 | 9084.3 | | 1271.81 |
| 22 | 7969.3 | | 2079.03 | 6350.7 | | 1574.54 | | 9084.2 | 2130.93 | | 3399.1 | 1414.88 | 4757.9 | | 1728.69 | 3420.2 | | 1232.1 |
| 23 | 3419.8 | | 2017.11 | 3420.1 | | 1488.17 | | 4091 | 2123.33 | | 3981.9 | 1376.32 | 6218.1 | | 1701.43 | 9041.4 | | 1187.88 |
| 24 | 9085 | | 1878.71 | 10136 | | 1482.86 | | 7339 | 1969.58 | | 4561.7 | 1360.35 | 3398.3 | | 1650.05 | 4091.4 | | 1133.33 |
| 25 | 5915.2 | | 1773.66 | 2698.9 | | 1446.51 | | 6204 | 1838.39 | | 6350.3 | 1324.99 | 3366.2 | | 1498.06 | 3367.1 | | 1132.62 |
| 26 | 4090.7 | | 1711.37 | 3366.7 | | 1256.26 | | 9040.7 | 1759.37 | | 10390.1 | 1194.07 | 2225.6 | | 1373.82 | 3981.8 | | 1005.22 |
| 27 | 7985.9 | | 1686.8 | 4090.4 | | 1235.03 | | 5393.9 | 1759.37 | | 5247.1 | 1183.35 | 3413.6 | | 1321.96 | 6353 | | 976.65 |
| 28 | 9040.2 | | 1606.77 | 3981.7 | | 1175.77 | | 3404.7 | 1697.07 | | 4090.6 | 1164.26 | 9375.9 | | 1186.81 | 3666.6 | | 971.37 |
| 29 | 3367.6 | | 1606.54 | 10105.7 | | 1158.14 | | 6960.8 | 1528.66 | | 2680.5 | 1111.17 | 9304.4 | | 1105.45 | 5187.6 | | 900.14 |
| 30 | 2681.7 | | 1568.26 | 5188 | | 1154.17 | | 3154.5 | 1433.39 | | 7338.2 | 1105.59 | 3980.8 | | 1073.95 | 2226.6 | | 886.27 |
| 31 | 5187.9 | | 1483.34 | 10937.8 | | 1130.97 | | 5465.9 | 1364.44 | | 4450.1 | 1026.86 | 2976.7 | | 1018.08 | 3471 | | 884.48 |
| 32 | 2281.2 | | 1405.69 | 6220.5 | | 1059.54 | | 3960 | 1330.2 | | 2225.9 | 1003 | 2525.7 | | 975.39 | 2281.6 | | 867.28 |
| 33 | 5319.9 | | 1329.2 | 10509.6 | | 1038.03 | | 2688.4 | 1293.05 | | 3472.9 | 936.7 | 2280.8 | | 974.92 | 10139.6 | | 815.08 |
| 34 | 5247 | | 1254.31 | 3667.1 | | 917.26 | | 5200.6 | 1217.8 | | 5186.4 | 929.92 | 2680.7 | | 929.89 | 10392.4 | | 744.37 |
| 35 | 5466.3 | | 1193.69 | 5060.9 | | 898.91 | | 5247.1 | 1213.4 | | 10511.1 | 889.87 | 2026.1 | | 915.96 | 5061.2 | | 738.17 |
| 36 | 2977.6 | | 1147.69 | 5459.3 | | 887.77 | | 3690.8 | 1211.97 | | 3450.1 | 845.92 | 9026.5 | | 908.22 | 5248.1 | | 737.38 |
| 37 | 10137.9 | | 1143.94 | 2226.7 | | 874 | | 5044.5 | 1076.39 | | 2280.8 | 817.63 | 5512.7 | | 898.64 | 3156.7 | | 717.95 |
| 38 | 3156.4 | | 1135.91 | 5247.1 | | 842.11 | | 6640.2 | 1049.31 | | 10936.8 | 812.58 | 2956.4 | | 879.17 | 5466.1 | | 662.6 |
| 39 | 3666.1 | | 1135 | 3155.4 | | 825.51 | | 10106.7 | 1044.17 | | 3154.6 | 807.52 | 3163.7 | | 835.4 | 2957.7 | | 632.1 |
| 40 | 5060.1 | | 1109.09 | 3470.2 | | 766.23 | | 2984.8 | 1005.31 | | 5516 | 794.1 | 3470 | | 828.29 | 5744.2 | | 632.05 |

Table S3. Cont.

|  | C13 | | F44 | | 8601 | | 94A150 | | 94A140 | | FD62 | |
| --- | --- | --- | --- | --- | --- | --- | --- | --- | --- | --- | --- | --- |
| No | m/z | Intens. | m/z | Intens. | m/z | Intens. | m/z | Intens. | m/z | Intens. | m/z | Intens. |
| 41 | 5515.7 | 1092.2 | 5045.8 | 685.66 | 3666.5 | 1002.3 | 2956.5 | 727.99 | 7796.9 | 784.22 | 10105.1 | 528.45 |
| 42 | 6350.2 | 1029.74 | 2957.1 | 640.4 | 2254.5 | 983.94 | 5462.5 | 727.11 | 9068.7 | 738.67 | 2379 | 502.42 |
| 43 | 8832 | 996.34 | 2280.8 | 632.94 | 6991 | 881.97 | 5058.6 | 686.02 | 10120.2 | 725.98 | 7056.2 | 491.02 |
| 44 | 10390 | 995.23 | 7482.1 | 609.34 | 10418 | 867.24 | 2378.7 | 625.62 | 3543 | 683.26 | 6153.6 | 447.45 |
| 45 | 3982.2 | 876.63 | 7056.4 | 600.51 | 10951.6 | 859.33 | 2330.6 | 590.65 | 10370.2 | 507.62 | 7200.2 | 438.13 |
| 46 | 2226.2 | 759.97 | 6152.8 | 539.91 | 6160.1 | 765.78 | 3666.3 | 469.83 | 10487.6 | 470.39 | 4667.4 | 437.18 |
| 47 | 5743.6 | 743.23 | 5742.9 | 532.71 | 3471.7 | 750.62 | 7091.3 | 438.68 | 10232.6 | 451.93 | 10938.7 | 421.85 |
| 48 | 10106.4 | 714.63 | 8071.3 | 530.77 | 3493.3 | 733.94 | 7482.4 | 410.57 | 10922.7 | 380.46 | 7483.4 | 413.39 |
| 49 | 10952.8 | 713.57 | 7192.1 | 520.63 | 7497.1 | 677.19 | 5357 | 373.72 | 8865.4 | 330.81 | 10511 | 403.09 |
| 50 | 7484.1 | 683.03 | 11507 | 481.66 | 7199.7 | 664.5 | 7746.3 | 356.42 | 9753.2 | 300.54 | 8064.8 | 375.13 |

m/z - intensity values of top 50 major peaks were listed. It includes one M1 type isolate(C13), one M2 type isolate(F44) and four M3 type isolates(8601, 94A150, 94A140, FD62).
