# Supplementary material for: Identification and Cluster Analysis of Streptococcus pyogenes by MALDI-TOF Mass Spectrometry
Source: PLoS One. 2012 Nov 7;7(11):e47152. doi: 10.1371/journal.pone.0047152 (PMC3492366; doi:10.1371/journal.pone.0047152)
Supplement: Table S4 — Peaklist for M4, M8 type isolates. m/z – intensity values of top 50 major peaks were listed. It includes two M4 type isolates (8602,8635) and four M8 type isolates (8609, 8614, 8639, 8645). (DOCX) [file pone.0047152.s006.docx]

Table S4. Peaklist for M4, M8 type isolates.

|  | | 8602 | | | 8635 | | | 8609 | | | 8614 | | 8639 | | 8645 | |
| --- | --- | --- | --- | --- | --- | --- | --- | --- | --- | --- | --- | --- | --- | --- | --- | --- |
| No | m/z | | Intens. | m/z | | Intens. | m/z | | Intens. | m/z | | Intens. | m/z | Intens. | m/z | Intens. |
| 1 | 9530.4 | | 18530.72 | 4577.1 | | 12702.97 | 6835 | | 18514.05 | 4563.8 | | 18732.75 | 4451.7 | 17941.26 | 9531.3 | 11190.71 |
| 2 | 6834.3 | | 16961.57 | 6833.8 | | 12050.34 | 4452.2 | | 15109.97 | 6838.4 | | 18007.9 | 6834.1 | 13647.17 | 4453 | 9272.57 |
| 3 | 4577.3 | | 16589.53 | 9529.4 | | 11698.46 | 4561.9 | | 13706.88 | 9535.9 | | 14469.77 | 5362.5 | 12153.71 | 5364.4 | 9097.14 |
| 4 | 4561.4 | | 14043.68 | 4561.3 | | 10504.23 | 5363.4 | | 13148.39 | 4453.6 | | 13618.54 | 4561.2 | 11755.09 | 6315.1 | 5423.57 |
| 5 | 6313.5 | | 10439.47 | 6313.3 | | 7960.03 | 6738.2 | | 11811.25 | 5365.9 | | 12063.34 | 6737 | 10339.54 | 6739.3 | 5259.57 |
| 6 | 6737.6 | | 10333.27 | 6737.3 | | 6770.97 | 6899.9 | | 9454.54 | 6741.6 | | 9649.54 | 6801.1 | 9990.54 | 6835.5 | 4945.66 |
| 7 | 6898.6 | | 8902.01 | 6899.1 | | 6012.37 | 6314.1 | | 9372.8 | 6317.2 | | 9329.36 | 6312.6 | 9055.09 | 6846 | 4891.74 |
| 8 | 4592.5 | | 8323.66 | 4592 | | 5217.63 | 9531.2 | | 8882.89 | 6805.3 | | 8621.4 | 5956.5 | 8106.26 | 6803 | 4366.54 |
| 9 | 5378.6 | | 7642.97 | 4605.2 | | 4629.89 | 6800.9 | | 8482.99 | 4579.3 | | 7635.12 | 9528.6 | 7720.26 | 5958.2 | 4168.91 |
| 10 | 8189.9 | | 6358.02 | 5377.4 | | 4447.09 | 5957.9 | | 8137.69 | 6902.3 | | 7525.36 | 6899 | 6022.57 | 4759.9 | 3833.86 |
| 11 | 4514 | | 6099 | 4513.7 | | 4345.2 | 4577.1 | | 7311.72 | 5960.6 | | 7414.86 | 6945.4 | 5902.4 | 7971.4 | 3062.97 |
| 12 | 6381.5 | | 5619.51 | 8189.3 | | 4323.23 | 8190.7 | | 6479.26 | 8194.7 | | 5938.38 | 8188.9 | 5585.34 | 8191.3 | 2820.06 |
| 13 | 4758.4 | | 5592.44 | 6381.4 | | 4315.43 | 5378.6 | | 6300.51 | 4593.7 | | 5294.44 | 7968.3 | 4052.49 | 6946 | 2774.03 |
| 14 | 4451.2 | | 5423.4 | 6800.1 | | 3762.94 | 6946.1 | | 5619.61 | 6949.1 | | 4863.84 | 5914.2 | 3796.2 | 7340.8 | 2700.14 |
| 15 | 5363.1 | | 5240.18 | 4451.7 | | 3588.51 | 4589.2 | | 5217.72 | 6223.8 | | 4719.69 | 6219.5 | 2589.63 | 4562.3 | 2261.43 |
| 16 | 5972.9 | | 5028.72 | 4758.2 | | 3586.51 | 4759.3 | | 3872.48 | 7974.6 | | 3726.26 | 3419.4 | 2587.91 | 6901.8 | 1627.29 |
| 17 | 5957.6 | | 4558.49 | 5972.1 | | 3255.74 | 4515.2 | | 3579.29 | 7343.7 | | 3302.64 | 4512.7 | 2563.29 | 5917.3 | 1544.8 |
| 18 | 7986.3 | | 4447.44 | 7339.6 | | 3010.03 | 3420.5 | | 3569.53 | 4517.2 | | 2947.3 | 7338.5 | 2394.91 | 3421 | 1504.94 |
| 19 | 7338.9 | | 3712.28 | 4484.2 | | 2909.23 | 7986.3 | | 3451.82 | 4761 | | 2867.88 | 5319.5 | 2306.74 | 9085.4 | 1464.8 |
| 20 | 5930.4 | | 3375.68 | 5362.2 | | 2841.23 | 7338.2 | | 3242.34 | 5917.8 | | 2768.15 | 4758.7 | 2239.83 | 2681.3 | 1426.37 |
| 21 | 9084.7 | | 2969.05 | 4499.3 | | 2781.43 | 5915.4 | | 3216.43 | 5320.4 | | 2539.05 | 9039.4 | 2146.51 | 4536.9 | 1403.31 |
| 22 | 3419.5 | | 2851.17 | 5957.6 | | 2780.86 | 7970 | | 3156.65 | 9089.7 | | 2296.57 | 4090.6 | 2129.29 | 4515.7 | 1376.8 |
| 23 | 9039.4 | | 2736.19 | 7985.2 | | 2699.74 | 3368 | | 2684.84 | 9044.1 | | 2129.27 | 9082.5 | 2043.2 | 9041.6 | 1296.94 |
| 24 | 4090.1 | | 2222.63 | 4467 | | 2645.83 | 6220 | | 2658.87 | 3422.1 | | 1800 | 3366.6 | 1973.94 | 3366.6 | 1179.14 |
| 25 | 3366.7 | | 1932.72 | 6960.8 | | 2096.54 | 5318.8 | | 2538.7 | 10145.5 | | 1644.08 | 2680.8 | 1827.31 | 6221.5 | 1167.37 |
| 26 | 10418.6 | | 1782.66 | 3420.5 | | 2049.6 | 2681.9 | | 2446.37 | 10396.4 | | 1409.34 | 5246.5 | 1420.29 | 4091.7 | 1155.57 |
| 27 | 10138.4 | | 1776.09 | 5930.3 | | 2037.54 | 4090.7 | | 2399.27 | 2682.9 | | 1369.72 | 5460.1 | 1383.34 | 3982.2 | 1152.83 |
| 28 | 5317.2 | | 1744.73 | 9039.3 | | 1888.43 | 9038.8 | | 2223.57 | 5518.6 | | 1278.88 | 2225.6 | 1328.03 | 3667.8 | 1105.66 |
| 29 | 5201.1 | | 1652.12 | 9082.8 | | 1679.66 | 9084.9 | | 2213.41 | 5465.5 | | 1269.17 | 2976.4 | 1265.51 | 5188.8 | 1096.23 |
| 30 | 5245.2 | | 1508.25 | 4089.9 | | 1547.97 | 5460.6 | | 1669.23 | 3368.8 | | 1255.23 | 3155.2 | 1234.74 | 2226.8 | 1077.09 |
| 31 | 5516.6 | | 1398.72 | 3366.5 | | 1319.37 | 5187.5 | | 1523.86 | 4091.7 | | 1095.88 | 10135.5 | 1165.03 | 4595.4 | 962.51 |
| 32 | 2288.8 | | 1370.67 | 3154.1 | | 1125.14 | 3666.3 | | 1514.65 | 5190.2 | | 1026.05 | 3666.4 | 1095.29 | 3471.9 | 926.86 |
| 33 | 2689.3 | | 1349.79 | 3667.3 | | 1068.37 | 3447 | | 1484.12 | 5247.2 | | 1009.04 | 5185.8 | 1020.63 | 10136.9 | 903.17 |
| 34 | 3989 | | 1347.34 | 5200.1 | | 1054.8 | 3470.8 | | 1481.86 | 2282.4 | | 955.85 | 3979.5 | 1014.86 | 10107.1 | 819.06 |
| 35 | 5061.9 | | 1316.02 | 2688.9 | | 1047.03 | 2978.1 | | 1456.32 | 10514.1 | | 951.91 | 5061.5 | 983.89 | 5061.5 | 818.6 |
| 36 | 3666.2 | | 1290.39 | 5061 | | 1038.91 | 3989 | | 1378.5 | 3668.3 | | 934.37 | 10508.2 | 933.63 | 10393.5 | 807.14 |
| 37 | 3154.1 | | 1121.62 | 10135.1 | | 958.63 | 5245.4 | | 1351.44 | 2228.2 | | 857.09 | 10935.3 | 924.91 | 5459.9 | 791.51 |
| 38 | 3448.3 | | 1088.82 | 10416.9 | | 943.63 | 10137.8 | | 1339.67 | 5064 | | 853.87 | 10388.4 | 895 | 3155.6 | 784.09 |
| 39 | 5458.7 | | 1044.69 | 5316.1 | | 937.37 | 5062 | | 1311.56 | 10945.9 | | 851.12 | 2280.3 | 844.97 | 2977.4 | 782.34 |
| 40 | 10510.9 | | 1008.11 | 5246.8 | | 926.77 | 2225.9 | | 1309.27 | 2978 | | 826.26 | 10104.9 | 751.11 | 5248.9 | 776.71 |

Table S4. Cont.

|  | 8602 | | 8635 | | 8609 | | 8614 | | 8639 | | 8645 | |
| --- | --- | --- | --- | --- | --- | --- | --- | --- | --- | --- | --- | --- |
| No | m/z | Intens. | m/z | Intens. | m/z | Intens. | m/z | Intens. | m/z | Intens. | m/z | Intens. |
| 41 | 3188.5 | 938.29 | 3989 | 920.34 | 5515.5 | 1307.24 | 3643.4 | 758.41 | 5513.5 | 721.8 | 8887.7 | 700.03 |
| 42 | 2755.8 | 916.99 | 5530.7 | 883.83 | 10393.4 | 1190.55 | 3157.9 | 751.57 | 7483.7 | 605 | 6168.5 | 670.57 |
| 43 | 2976.9 | 897.48 | 5459.9 | 878.49 | 3154.7 | 1181.49 | 3989.7 | 689.32 | 2755.8 | 520.8 | 10938.2 | 588.31 |
| 44 | 10939.4 | 870.6 | 5514.6 | 861 | 2280.2 | 1097.49 | 2756.7 | 618.07 | 5743.9 | 421.03 | 5745 | 546.09 |
| 45 | 2225.7 | 770.47 | 2280.1 | 828.8 | 2244.4 | 916.91 | 11515.1 | 212.64 | 7196.4 | 304.69 | 10512.5 | 530.54 |
| 46 | 7200.7 | 765.93 | 2680.2 | 744.14 | 10510 | 837.07 | 12366.9 | 169.38 | 9865.5 | 228.11 | 7484.3 | 525.23 |
| 47 | 4312.7 | 752.01 | 2976.8 | 592.03 | 2755.8 | 781.91 | 12170 | 116.34 | 8828.1 | 221.17 | 7201.6 | 507.37 |
| 48 | 7496.3 | 542.43 | 2754.9 | 556.4 | 10938.6 | 758.52 | 13337.2 | 105.39 | 11501.2 | 205.49 | 2280.3 | 487.31 |
| 49 | 11531.6 | 317.39 | 10509.8 | 486.83 | 2041.7 | 668.58 | 15019.7 | 88.1 | 12350.8 | 107.69 | 8070 | 413.2 |
| 50 | 12348.8 | 209.04 | 10937.3 | 478.86 | 11506.4 | 165.85 | 14153.8 | 57.53 | 15003.5 | 93.23 | 7057.9 | 393.94 |

m/z - intensity values of top 50 major peaks were listed. It includes two M4 type isolates (8602,8635) and four M8 type isolates (8609, 8614, 8639, 8645).
