# Supplementary material for: Identification and Cluster Analysis of Streptococcus pyogenes by MALDI-TOF Mass Spectrometry
Source: PLoS One. 2012 Nov 7;7(11):e47152. doi: 10.1371/journal.pone.0047152 (PMC3492366; doi:10.1371/journal.pone.0047152)
Supplement: Table S5 — Peaklist for M12 type isolates (part 1). m/z – intensity values of top 50 major peaks were listed. It includes six isolates of M12 type (8604, 8605, 8606, 8611, 8613, 8621). (DOCX) [file pone.0047152.s007.docx]

Table S5. Peaklist for M12 type isolates (part 1).

|  | | 8604 | | 8605 | | 8606 | | | 8611 | | | 8613 | | | 8621 | | |
| --- | --- | --- | --- | --- | --- | --- | --- | --- | --- | --- | --- | --- | --- | --- | --- | --- | --- |
| No | m/z | | Intens. | m/z | Intens. | | m/z | Intens. | | m/z | Intens. | | m/z | Intens. | | m/z | Intens. |
| 1 | 9529.9 | | 14756.97 | 4544 | 20660.24 | | 4545 | 27148.08 | | 4544.9 | 24279.06 | | 4557.9 | 38657.81 | | 4543.5 | 19804.83 |
| 2 | 4451.7 | | 8851.89 | 4572.1 | 14118.08 | | 4572.9 | 22593.78 | | 4573 | 19940.43 | | 4529.9 | 31377.19 | | 4571.3 | 10322.99 |
| 3 | 4542.6 | | 8352.71 | 9531.9 | 12920.72 | | 9533.5 | 17832.05 | | 9533.5 | 13093.24 | | 4452.6 | 14017.42 | | 9529.7 | 10195.15 |
| 4 | 5361.8 | | 7767.24 | 6835.7 | 12786.22 | | 4453.5 | 15072.81 | | 6836.8 | 10670.51 | | 6835.6 | 13265.15 | | 4452 | 9442.93 |
| 5 | 6313.4 | | 5466.17 | 4559.7 | 12221.51 | | 6837.1 | 11823.72 | | 4559.8 | 10576.61 | | 6914.2 | 12986.35 | | 6834.4 | 8508.21 |
| 6 | 4758.3 | | 4775.35 | 4452.6 | 10511.82 | | 6916 | 10981.5 | | 4453.5 | 10495.46 | | 4572.6 | 12708.52 | | 5363.1 | 7497.16 |
| 7 | 6737.5 | | 4660.76 | 6914.8 | 9648.75 | | 6740.2 | 10703.98 | | 5365 | 8894.77 | | 5363.7 | 11065.71 | | 6313.7 | 5742.67 |
| 8 | 6800.7 | | 4655.82 | 6314.8 | 8536.78 | | 5364.8 | 10028.71 | | 6915.4 | 8852.83 | | 9516.2 | 10342.01 | | 6913.3 | 5724.92 |
| 9 | 5912.9 | | 4571.95 | 6738.7 | 8318.54 | | 6315.9 | 9149.68 | | 6740.1 | 7150.42 | | 6738.7 | 10214.57 | | 6801.1 | 5425.23 |
| 10 | 6844.3 | | 4284.7 | 4587.2 | 7508.67 | | 6804 | 8434.74 | | 6315.8 | 6797.89 | | 6802.3 | 9452.95 | | 6736.9 | 5379.03 |
| 11 | 7969.8 | | 3326.88 | 5363.4 | 7496.38 | | 5915.2 | 6608.81 | | 6802.1 | 6132.25 | | 6314.7 | 8017.77 | | 4758.5 | 4912.96 |
| 12 | 4571.2 | | 2623.19 | 5378.4 | 5147.87 | | 8192.6 | 5810.86 | | 4759.6 | 4288.91 | | 6946.6 | 6270.81 | | 5913.1 | 4009.87 |
| 13 | 8189.2 | | 2452.3 | 4759.6 | 4726.33 | | 6948.3 | 5136.1 | | 6944.8 | 4130.18 | | 5913.9 | 6066.05 | | 6946.4 | 3703.16 |
| 14 | 6945 | | 2450.93 | 8191.6 | 4676.44 | | 4760.4 | 4505.56 | | 8192.8 | 4116.76 | | 8190.9 | 5590.66 | | 7969.6 | 3199.79 |
| 15 | 7338.1 | | 2182.77 | 5914.8 | 3752.34 | | 7972.9 | 4413.69 | | 5380.7 | 3979.76 | | 5929.2 | 4200.36 | | 6365.3 | 3058.22 |
| 16 | 6219.9 | | 2145.37 | 5929.5 | 3589.37 | | 5930.4 | 3905.34 | | 5915.2 | 3639.35 | | 6367 | 3587.42 | | 8189.6 | 2894.21 |
| 17 | 6913 | | 1642.12 | 6235.4 | 3487.97 | | 6367.4 | 3502.2 | | 5931.2 | 2783.85 | | 7969.8 | 3123.54 | | 3419.3 | 2508.63 |
| 18 | 2680.2 | | 1309.56 | 7341 | 3356.17 | | 5957.8 | 2937.13 | | 6367.5 | 2682.99 | | 5954.9 | 3063.85 | | 6233.8 | 2272.28 |
| 19 | 6351.6 | | 1267.04 | 7987.3 | 3136.48 | | 6236.5 | 2774.56 | | 6235.6 | 2465.66 | | 5317.4 | 2491.28 | | 5954.9 | 1907.66 |
| 20 | 9084.3 | | 1260.72 | 3420.1 | 2552.71 | | 7340.6 | 2731.5 | | 3421.3 | 2373.95 | | 7339.7 | 2244.33 | | 7339.2 | 1832.53 |
| 21 | 3418.5 | | 1241.18 | 5957 | 2421.61 | | 9042.9 | 2547.46 | | 7987.9 | 2173.79 | | 5544.7 | 2107.86 | | 2271.6 | 1700.21 |
| 22 | 4090.1 | | 1164.11 | 6366.9 | 2388.39 | | 9085.9 | 2498.22 | | 5957.1 | 2160.55 | | 9040.1 | 2083.12 | | 3367.1 | 1636.53 |
| 23 | 3366.5 | | 1141.8 | 9086.9 | 2051.96 | | 3421.7 | 2105.87 | | 7342 | 2148.78 | | 4750.3 | 2047.09 | | 3398.8 | 1596.26 |
| 24 | 5186.3 | | 1060.01 | 9040.7 | 2035.14 | | 5322.8 | 1941.33 | | 5319.7 | 1840.35 | | 9085.3 | 1976.73 | | 2680.6 | 1582.86 |
| 25 | 3398.6 | | 1028.17 | 3367.1 | 1820.76 | | 10139.9 | 1930.01 | | 7971.4 | 1836.75 | | 6234.3 | 1970.67 | | 4090.8 | 1566.88 |
| 26 | 5246.2 | | 1005.94 | 4091.3 | 1720.68 | | 10395.6 | 1846.21 | | 2272.7 | 1604.21 | | 3419.1 | 1945.3 | | 3454 | 1480.7 |
| 27 | 3981.1 | | 999.25 | 5188.1 | 1475.71 | | 3367.4 | 1731.39 | | 2682 | 1536.6 | | 2278.7 | 1831.67 | | 5186.4 | 1433.24 |
| 28 | 9037.5 | | 970.37 | 5246.7 | 1415.35 | | 5545.7 | 1491.51 | | 4091.3 | 1531.02 | | 4091.7 | 1811.28 | | 3981.2 | 1376.62 |
| 29 | 5061 | | 964.44 | 3454.9 | 1410.62 | | 4091.7 | 1454.56 | | 9087.2 | 1513.95 | | 3367.3 | 1536.82 | | 9083.7 | 1335.73 |
| 30 | 5459.9 | | 953.93 | 3667.5 | 1295.63 | | 5188.8 | 1439.94 | | 2286.7 | 1483.97 | | 3400.2 | 1508.44 | | 3470.3 | 1304.99 |
| 31 | 3665.6 | | 948.14 | 10137.1 | 1265.68 | | 2682.1 | 1270.07 | | 9043.7 | 1451.47 | | 5867.3 | 1459.48 | | 5247.3 | 1231.02 |
| 32 | 3469.6 | | 888.54 | 10393.8 | 1262.89 | | 5461.7 | 1191.05 | | 3368.8 | 1327.71 | | 10138.9 | 1348.52 | | 9038.8 | 1216.43 |
| 33 | 10138 | | 852.95 | 2280.5 | 1204.12 | | 5062.8 | 1176.02 | | 3454.5 | 1273.02 | | 5461.9 | 1332.12 | | 3666.2 | 1099.05 |
| 34 | 10391.2 | | 844.78 | 3989.2 | 1163.76 | | 2285.4 | 1162.38 | | 5546.3 | 1165.22 | | 3454.8 | 1198.39 | | 2225.6 | 1088.79 |
| 35 | 8831.1 | | 824.16 | 5061.6 | 1153.73 | | 10940.8 | 1152.21 | | 5189 | 1156.13 | | 2680.9 | 1176.93 | | 2976.2 | 1087.44 |
| 36 | 2955.1 | | 823.3 | 5460.9 | 1143.49 | | 3454.5 | 1144.7 | | 5460.9 | 1060.27 | | 5186.9 | 1149.8 | | 2955.5 | 1068.99 |
| 37 | 5756.3 | | 784.3 | 3155.4 | 1134.09 | | 3990.9 | 1097.99 | | 5248.3 | 1048.32 | | 2976.7 | 1056.52 | | 3155.3 | 1045.42 |
| 38 | 3155.5 | | 780.45 | 2688.6 | 1082.82 | | 10511.8 | 1082.53 | | 3667.6 | 1037.8 | | 10389.8 | 1013.78 | | 5060.7 | 1038.6 |
| 39 | 2026.7 | | 773.9 | 5544.1 | 1036.67 | | 2228 | 1043.66 | | 2226.6 | 990.26 | | 3982.5 | 943.21 | | 5459.6 | 994.88 |
| 40 | 2225 | | 754.36 | 2227 | 917.28 | | 2956.5 | 903.57 | | 3156.8 | 910.61 | | 10511.3 | 916.62 | | 10391 | 942.4 |

Table S5. Cont.

|  | 8604 | | 8605 | | 8606 | | 8611 | | 8613 | | 8621 | |
| --- | --- | --- | --- | --- | --- | --- | --- | --- | --- | --- | --- | --- |
| No | m/z | Intens. | m/z | Intens. | m/z | Intens. | m/z | Intens. | m/z | Intens. | m/z | Intens. |
| 41 | 10106.5 | 743.61 | 2977.2 | 846.71 | 3156.1 | 882.09 | 2756.9 | 886.37 | 5756.2 | 905.98 | 3180.9 | 895.87 |
| 42 | 5045.3 | 715 | 10512.8 | 840.12 | 3667.3 | 876.61 | 10140.3 | 854.11 | 10938.4 | 821.56 | 2755.9 | 766.97 |
| 43 | 6152.1 | 674.95 | 2756.6 | 734.53 | 5757.6 | 871.99 | 3987.4 | 825.71 | 5059.3 | 818.26 | 10133.2 | 758.35 |
| 44 | 2270.7 | 638.53 | 10941.1 | 725.34 | 2755.4 | 656.05 | 2977.7 | 736.28 | 3641.2 | 817.7 | 5754.8 | 735.57 |
| 45 | 10938.3 | 510.36 | 5755.9 | 724.04 | 11536.3 | 585.61 | 10392.4 | 726.54 | 2962.2 | 797.84 | 7482.6 | 580.58 |
| 46 | 2377.5 | 501.72 | 7489.3 | 488.2 | 8835.3 | 439.87 | 5059 | 667.06 | 3156.8 | 770.32 | 7199.8 | 523.95 |
| 47 | 10509.7 | 492.12 | 11535.7 | 321.8 | 12333.5 | 277.76 | 5756.6 | 647.01 | 3666.2 | 756.98 | 10509.3 | 504.06 |
| 48 | 7055.3 | 487.87 | 12337.3 | 144.14 | 12171.3 | 209.66 | 10512.8 | 557.55 | 2754.4 | 648.82 | 10938.6 | 423.48 |
| 49 | 9869.4 | 426.96 | 12175.3 | 106.02 | 13345.4 | 169.65 | 10943.6 | 445.21 | 4027 | 616.81 | 8829.6 | 410.01 |
| 50 | 12172.1 | 334.16 | 13332.6 | 93.83 | 13273.7 | 141.25 | 11537 | 164.24 | 11536.9 | 292.61 | 7746.4 | 395.94 |

m/z - intensity values of top 50 major peaks were listed. It includes six isolates of M12 type (8604, 8605, 8606, 8611, 8613, 8621).
