# Supplementary material for: Identification and Cluster Analysis of Streptococcus pyogenes by MALDI-TOF Mass Spectrometry
Source: PLoS One. 2012 Nov 7;7(11):e47152. doi: 10.1371/journal.pone.0047152 (PMC3492366; doi:10.1371/journal.pone.0047152)
Supplement: Table S6 — Peaklist for M12 type isolates (part 2). m/z – intensity values of top 50 major peaks were listed. It includes six isolates of M12 type (94A148, B09, B26, C26, C30, E205). (DOCX) [file pone.0047152.s008.docx]

Table S6. Peaklist for M12 type isolates (part 2).

|  | 94A148 | | B09 | | B26 | | C26 | | C30 | | E205 | |
| --- | --- | --- | --- | --- | --- | --- | --- | --- | --- | --- | --- | --- |
| No | m/z | Intens. | m/z | Intens. | m/z | Intens. | m/z | Intens. | m/z | Intens. | m/z | Intens. |
| 1 | 9531.5 | 10784.02 | 4544.3 | 9009 | 4560.6 | 26397.24 | 4532.6 | 13364.57 | 4532.8 | 10295.71 | 4544 | 16975.04 |
| 2 | 4544.2 | 10175.8 | 4452.7 | 4916 | 4532.6 | 20103.57 | 4451.4 | 12977.14 | 9527.6 | 7145.2 | 9530.4 | 12372.13 |
| 3 | 4452.8 | 9986.83 | 4572.2 | 4054 | 9528.2 | 17371.35 | 6832.1 | 9785.69 | 4451.2 | 6078.66 | 4452.6 | 10847.88 |
| 4 | 5364 | 9399.79 | 6836 | 2786 | 4451.1 | 14046.29 | 6736.4 | 9114.09 | 4547.9 | 5561.89 | 6835 | 10166.98 |
| 5 | 4572.4 | 6585.29 | 6914.9 | 2406 | 6832.8 | 13652.83 | 6841.8 | 9069.57 | 6832.9 | 5487.71 | 4571 | 9537.07 |
| 6 | 6835.6 | 6402.94 | 6803.5 | 2073 | 6840.2 | 11428.25 | 6799.5 | 8417.03 | 6312.3 | 4969.74 | 4559.8 | 9243.04 |
| 7 | 6738.9 | 6272.07 | 6315.7 | 1941 | 4547.9 | 11403.46 | 5361.8 | 8133.06 | 6842.3 | 4611.46 | 6738.1 | 8707.89 |
| 8 | 6314.6 | 6065.04 | 6740 | 1897 | 5361.9 | 10889.84 | 4548.2 | 7173.77 | 6736.4 | 4304.77 | 6314.4 | 7210.16 |
| 9 | 6802.5 | 5609.36 | 5364.2 | 1579 | 6736.2 | 10735.1 | 6312.4 | 6675.66 | 5362.2 | 4021.49 | 6801.7 | 6820.87 |
| 10 | 5914.1 | 4736.91 | 6948.4 | 1444 | 6312.4 | 10060.82 | 6911.8 | 5844.8 | 6799.6 | 3475.03 | 6913.8 | 6760.49 |
| 11 | 6914 | 3558.15 | 5914.6 | 1189 | 6800.2 | 9029.3 | 5911.8 | 4853.06 | 4575.7 | 3300.26 | 6816 | 6018.55 |
| 12 | 6947.7 | 3193.44 | 6367.2 | 1080 | 4575.2 | 8290.08 | 4573.9 | 4476.2 | 6816.2 | 3242.63 | 8190.7 | 4604.27 |
| 13 | 8191.2 | 3085.45 | 9534.2 | 1052 | 6911.3 | 8036.7 | 5927.2 | 4085.8 | 6911.7 | 3236.86 | 5913.3 | 4593.97 |
| 14 | 7971.1 | 2861.89 | 8192.3 | 743 | 8187.8 | 6480.63 | 5376.8 | 4045.91 | 6218.8 | 2558.17 | 6945.4 | 3738.17 |
| 15 | 4759.5 | 2120.45 | 2272.2 | 638 | 5911.9 | 5813.52 | 8187.3 | 3983.06 | 8188.5 | 2479.94 | 5363.2 | 3601.9 |
| 16 | 7340 | 1946.12 | 5956.7 | 617 | 4514.6 | 5403.37 | 6945.3 | 3795.69 | 4513.3 | 2446.11 | 5930 | 3486.61 |
| 17 | 6220.3 | 1844.86 | 3420.5 | 595 | 5377.1 | 5322.04 | 9526.9 | 3703.97 | 5377.1 | 2291.57 | 6221 | 3046.6 |
| 18 | 5319 | 1668.74 | 3367.4 | 571 | 5927.7 | 4889.35 | 4516.7 | 3427.09 | 5911.8 | 2167.49 | 4759.3 | 2608.24 |
| 19 | 6352.2 | 1402.22 | 3399.2 | 561 | 6945.7 | 4850.63 | 4596.5 | 3326.2 | 6945.2 | 2012.43 | 5956.1 | 1959.41 |
| 20 | 9040.8 | 1197.85 | 3455.1 | 503 | 4595.7 | 4193.42 | 5318.8 | 2337.97 | 5926.4 | 1857.4 | 5379 | 1941.32 |
| 21 | 9086.3 | 1174.09 | 2225.3 | 495 | 7985.2 | 4073.27 | 5952.8 | 2224.74 | 4597.4 | 1715.54 | 3419.8 | 1689.2 |
| 22 | 2681.5 | 1171.39 | 5942.1 | 450 | 6218.2 | 4036.03 | 6218.5 | 2060.94 | 4758.2 | 1687.43 | 7971.8 | 1605.52 |
| 23 | 2329.2 | 1131.96 | 3156.2 | 438 | 4757.7 | 3713.72 | 5543 | 1357.26 | 7984.9 | 1400.14 | 9038.9 | 1544.46 |
| 24 | 3419.7 | 1011.06 | 4759 | 430 | 7968.9 | 3271.17 | 6351.1 | 1356.6 | 7336.9 | 1296.69 | 5543.6 | 1541.78 |
| 25 | 2458.9 | 1005.17 | 2680.5 | 418 | 5953.8 | 3075.93 | 3418.8 | 1285.34 | 6349.9 | 1251.37 | 6352 | 1534.76 |
| 26 | 2378.1 | 1003.78 | 4090.3 | 415 | 6349.9 | 2793.85 | 4089.8 | 1103.17 | 5952.8 | 1158.37 | 3366.5 | 1387.99 |
| 27 | 5545.2 | 962.89 | 2955.5 | 389 | 9082.5 | 2692.34 | 7984.2 | 1071.6 | 7967.6 | 1061.2 | 4090.5 | 1383.09 |
| 28 | 3368 | 960.34 | 2466.3 | 385 | 7337.8 | 2687.93 | 3366.6 | 1032.83 | 9081.1 | 1040.83 | 9083.7 | 1318.48 |
| 29 | 3399.1 | 908.99 | 2756.2 | 383 | 9038.9 | 2528.57 | 9036.1 | 1014.57 | 9038.1 | 1025.26 | 2271.7 | 807.23 |
| 30 | 5460.4 | 893.31 | 5320.5 | 365 | 3418.3 | 2131.76 | 2458.5 | 878.6 | 3419.2 | 995.26 | 3989.4 | 796.6 |
| 31 | 4091 | 886.48 | 3181.4 | 343 | 5541.6 | 1865.17 | 5456.8 | 790.4 | 5321.9 | 971.2 | 5320.3 | 786.75 |
| 32 | 2226.6 | 755.85 | 7972.6 | 333 | 10388.8 | 1728.96 | 2274 | 786.6 | 3366.3 | 928.94 | 3155.5 | 770.2 |
| 33 | 3981.5 | 739.51 | 3666.5 | 277 | 3366.4 | 1683.36 | 5245.8 | 750.34 | 4089.7 | 898.54 | 7339.9 | 764.4 |
| 34 | 5187.4 | 720 | 5244.9 | 273 | 10134.3 | 1682.75 | 7338.8 | 729.17 | 2274.9 | 632.34 | 5459 | 758.34 |
| 35 | 2924.6 | 706.08 | 3982.4 | 259 | 4088.9 | 1646.52 | 7968.7 | 727.86 | 3154.4 | 619.86 | 2957.2 | 717.92 |
| 36 | 3667.4 | 654.24 | 5188.6 | 245 | 5186.7 | 1347.29 | 2679.5 | 710.31 | 2678.8 | 602.8 | 5247 | 716.39 |
| 37 | 2273.4 | 639.84 | 5459.8 | 239 | 2680.3 | 1208.19 | 3641.8 | 669.74 | 5184.6 | 588.14 | 5186.8 | 692.41 |
| 38 | 3472.3 | 595.2 | 9044.4 | 206 | 2281.3 | 1197.32 | 4758 | 645.43 | 3987.4 | 582.89 | 2225.6 | 689.6 |
| 39 | 10141 | 587.94 | 7339.7 | 200 | 5246.4 | 1128.24 | 2224.7 | 637.63 | 5541.4 | 578.83 | 2681 | 652.26 |
| 40 | 5061 | 572.47 | 9088.5 | 195 | 5458.3 | 1078.52 | 3154.5 | 607.86 | 5459.8 | 569.51 | 5061.4 | 647.4 |

Table S6. Cont.

|  | 94A148 | | B09 | | B26 | | C26 | | C30 | | E205 | |
| --- | --- | --- | --- | --- | --- | --- | --- | --- | --- | --- | --- | --- |
| No | m/z | Intens. | m/z | Intens. | m/z | Intens. | m/z | Intens. | m/z | Intens. | m/z | Intens. |
| 41 | 10392.7 | 551.99 | 5059.7 | 193 | 10507.4 | 971.99 | 9081.5 | 523.26 | 10133.4 | 567.43 | 10134.9 | 585.24 |
| 42 | 2532.2 | 495.54 | 6233.7 | 193 | 5060.4 | 966.81 | 2975.9 | 496.8 | 10388.7 | 509.86 | 10938.1 | 577.68 |
| 43 | 3154.6 | 490.67 | 5753.9 | 134 | 3986.2 | 965.56 | 5184.2 | 472.14 | 5246.9 | 485.06 | 10392.2 | 555.04 |
| 44 | 9633.7 | 489.45 | 7484.8 | 76 | 3155.4 | 941.54 | 3984.6 | 411.89 | 2224.8 | 481.23 | 3667.4 | 504.72 |
| 45 | 2755.6 | 463.69 | 8067.9 | 65 | 3665.6 | 859.05 | 10934.3 | 393.66 | 3665.2 | 472.26 | 10509.2 | 488.76 |
| 46 | 3641.4 | 446.06 | 10513.7 | 61 | 10935.2 | 854.13 | 10131.9 | 231.34 | 2975 | 450.09 | 7482.2 | 290.71 |
| 47 | 10940.7 | 423.62 | 10943.4 | 60 | 2226 | 827.16 | 7483 | 194.94 | 5754.2 | 378.74 | 9868.7 | 171.93 |
| 48 | 10509.8 | 396.37 | 10112.4 | 52 | 2963.1 | 826.73 | 10506.8 | 181.57 | 5056 | 368 | 11526.3 | 133.2 |
| 49 | 3523.9 | 372.45 | 10392.1 | 44 | 3639.8 | 775.35 | 5056.2 | 178.6 | 10935.1 | 362.09 | 12168.4 | 62.92 |
| 50 | 2832 | 371.91 | 9871.9 | 28 | 5753.2 | 761.8 | 10388.3 | 178.4 | 10509 | 334.03 | 12343.1 | 57.26 |

m/z - intensity values of top 50 major peaks were listed. It includes six isolates of M12 type (94A148, B09, B26, C26, C30, E205).
