# Supplementary material for: Identification and Cluster Analysis of Streptococcus pyogenes by MALDI-TOF Mass Spectrometry
Source: PLoS One. 2012 Nov 7;7(11):e47152. doi: 10.1371/journal.pone.0047152 (PMC3492366; doi:10.1371/journal.pone.0047152)
Supplement: Table S7 — Peaklist for M12 type isolates ((part 3). m/z – intensity values of top 50 major peaks were listed. It includes six isolates of M12 type (J22, SZ–47, SZ–73, SZ–98, SZ–117, SZ–138). (DOCX) [file pone.0047152.s009.docx]

Table S7. Peaklist for M12 type isolates ((part 3).

|  | J22 | | SZ-47 | | SZ-73 | | SZ-98 | | SZ-117 | | SZ-138 | |
| --- | --- | --- | --- | --- | --- | --- | --- | --- | --- | --- | --- | --- |
| No | m/z | Intens. | m/z | Intens. | m/z | Intens. | m/z | Intens. | m/z | Intens. | m/z | Intens. |
| 1 | 4544.4 | 15550.72 | 4532.2 | 17678.51 | 4534.4 | 12702.39 | 9528.1 | 10552.11 | 4532.9 | 9897.8 | 4543.6 | 23726.83 |
| 2 | 4572.3 | 12569.71 | 4450.6 | 10918.34 | 4550.1 | 7130.24 | 4560.7 | 10473.8 | 4560.6 | 8392.2 | 4571.7 | 19346.17 |
| 3 | 9531.3 | 11173.71 | 9527.4 | 8582.57 | 4562.3 | 6982.93 | 4451 | 10326.06 | 9528.4 | 7818.69 | 9530.4 | 15894.83 |
| 4 | 4452.7 | 7331.36 | 5361.1 | 7930.77 | 9531.7 | 6770.28 | 4547.9 | 7525.91 | 4451.4 | 7064.54 | 4451.8 | 14072.03 |
| 5 | 4559 | 6791.37 | 6832.3 | 6859.71 | 6835.2 | 6060.81 | 6832.9 | 7068.86 | 6831.6 | 6825.71 | 6834.3 | 12343.31 |
| 6 | 6834.7 | 5919.11 | 6799.4 | 5500.14 | 4452.2 | 5664.28 | 6311.8 | 6711.26 | 4548.5 | 6371.29 | 4558.5 | 11328.63 |
| 7 | 5363.9 | 5849.98 | 6735.8 | 5441.91 | 6739 | 4528.58 | 6735.7 | 6231.37 | 6312.7 | 5499.03 | 6737.3 | 11159.06 |
| 8 | 6844.7 | 5615.96 | 6311.2 | 5304.91 | 6314.8 | 4260.2 | 6800.2 | 5938.09 | 6736.5 | 4657.63 | 6913.7 | 10836.17 |
| 9 | 6314.5 | 5378.33 | 4559.9 | 4747.34 | 5363.7 | 4127.52 | 6816 | 4646.4 | 6911.4 | 3842.63 | 6313.8 | 9750.31 |
| 10 | 6913.9 | 5282.52 | 5911.7 | 4647.03 | 6914 | 3596.71 | 5911.9 | 4426.46 | 6799.5 | 3827.06 | 5363.2 | 8630.4 |
| 11 | 6738.6 | 5177.58 | 6911 | 3726.46 | 6802.6 | 3256.09 | 6911.6 | 4293.74 | 4575.4 | 3553.74 | 6801.7 | 8365.34 |
| 12 | 4587.7 | 4647.72 | 7968 | 3694.57 | 4577 | 3162.16 | 8188.1 | 4186.29 | 6816.1 | 3538.14 | 4587.1 | 7824.71 |
| 13 | 6802.2 | 4594.57 | 6944.5 | 3314.03 | 6818.1 | 3162.12 | 4575.5 | 4173.91 | 6817.2 | 3498.14 | 6817.3 | 7514.74 |
| 14 | 6818.5 | 3667.16 | 8187.2 | 2872.86 | 5379.4 | 2586.46 | 5927.6 | 2983.71 | 5362.1 | 3319.11 | 8189.9 | 6592.83 |
| 15 | 8191 | 2785.72 | 6218.4 | 2641.37 | 8190.8 | 2179.13 | 6945.3 | 2829.09 | 8188.2 | 2779.29 | 5913.2 | 5716.57 |
| 16 | 5913.5 | 2785.07 | 4756.4 | 2237.91 | 6221.6 | 2106.66 | 7984.5 | 2779 | 4757.6 | 2757.46 | 5929.2 | 4735.51 |
| 17 | 4759.8 | 2752.05 | 6931.5 | 1783.66 | 5914.3 | 2097.57 | 7969.2 | 2603.83 | 6219 | 2491.49 | 6947 | 4591.09 |
| 18 | 6945.7 | 2647.38 | 5952 | 1568.03 | 4759.5 | 1878.07 | 6218.5 | 2266.94 | 5911.3 | 2343.23 | 5377.6 | 4261.31 |
| 19 | 5379 | 2575.51 | 4594.5 | 1514.49 | 6944.5 | 1850.07 | 5361.9 | 2166.77 | 5928.6 | 2029.86 | 7986.1 | 4137.71 |
| 20 | 5929.5 | 2043.62 | 7337 | 1400.2 | 5929 | 1803.51 | 9038.5 | 1839.43 | 6945.5 | 2006.77 | 7970.8 | 3086.34 |
| 21 | 6365.1 | 1753.85 | 3418.5 | 1296.37 | 3421.6 | 1348.5 | 4757.1 | 1769.66 | 5377 | 1832.57 | 5955 | 2630.34 |
| 22 | 7986.2 | 1641.38 | 5319.1 | 1264 | 7986.4 | 1222.62 | 5955.5 | 1694.4 | 7984.1 | 1665.66 | 9039.5 | 2568.66 |
| 23 | 5957.1 | 1567.81 | 3365.4 | 1201.94 | 7339.6 | 1202.35 | 9083.2 | 1556.4 | 3418.9 | 1460.49 | 7339.2 | 2534.8 |
| 24 | 7340.7 | 1563.4 | 5939.3 | 1163.11 | 5957.4 | 1083.02 | 6349.7 | 1272.37 | 7968.4 | 1293.69 | 9084.2 | 2415.94 |
| 25 | 7971.5 | 1461.99 | 6349.4 | 1158.66 | 3367.4 | 987.14 | 10136.2 | 1180.54 | 5955.1 | 1237.77 | 4758.9 | 2373.57 |
| 26 | 6234.6 | 1338.06 | 4088.8 | 1128.51 | 4091.3 | 961.88 | 4089.1 | 1047.09 | 9082.3 | 1165.43 | 6381 | 2117.74 |
| 27 | 3422.5 | 1331.63 | 9081.5 | 1074.97 | 5320.1 | 870.57 | 5376.7 | 1044.77 | 3365.8 | 1152.49 | 5318.2 | 1844.29 |
| 28 | 9041.4 | 1156.94 | 9037.9 | 1067.26 | 2680.4 | 813.44 | 10389.3 | 925.77 | 4089.7 | 1130.4 | 6250.8 | 1761.46 |
| 29 | 2681.5 | 1155.84 | 2680 | 954.23 | 9084.1 | 810.13 | 3397.3 | 917.6 | 9038.9 | 1122.46 | 10138 | 1647.89 |
| 30 | 4092.4 | 1131.12 | 3979.3 | 950.09 | 7970.3 | 797.99 | 3366.2 | 896.14 | 6351 | 1052.54 | 3420.5 | 1572.86 |
| 31 | 9084.1 | 1123.41 | 2265.1 | 927.06 | 9040.3 | 779.68 | 5545.5 | 879.23 | 3155.6 | 911.74 | 5543.5 | 1504.23 |
| 32 | 6342.1 | 1123.4 | 3155.2 | 859.14 | 2268 | 759.08 | 10936.6 | 714.83 | 5185.7 | 884.17 | 3367.5 | 1439.23 |
| 33 | 3368.7 | 1043.91 | 3470.2 | 802.4 | 2226.5 | 728.4 | 5243.9 | 711.77 | 2280.4 | 878.63 | 10391.3 | 1433.23 |
| 34 | 2272 | 955.67 | 5185.7 | 799.11 | 6354.7 | 715.67 | 10507.6 | 708.37 | 5244.6 | 839.2 | 4091.1 | 1386.06 |
| 35 | 5188.8 | 928.39 | 3453.6 | 770.14 | 5188.5 | 686 | 5060.1 | 671.89 | 3454.5 | 829.06 | 6340.9 | 1283.2 |
| 36 | 5543.5 | 781.48 | 2953.9 | 737.77 | 5544.4 | 681.76 | 3988.1 | 662.8 | 3988.1 | 809.83 | 10509.6 | 1104.8 |
| 37 | 3667.9 | 778.51 | 5243.8 | 708.31 | 3453.8 | 672.1 | 2265.5 | 612.83 | 7336.7 | 808.2 | 10938.2 | 1078.34 |
| 38 | 2226.3 | 766.04 | 10135.2 | 705.14 | 3989.4 | 621.6 | 5460.3 | 611.71 | 10134.8 | 806.34 | 5245.6 | 978.86 |
| 39 | 3156.8 | 744.02 | 2225.5 | 698.63 | 5246.6 | 591.24 | 2225.7 | 536.97 | 5059.1 | 728.54 | 5461.4 | 941.17 |
| 40 | 2756.8 | 727.21 | 5460.1 | 694.97 | 3667.4 | 570.48 | 5185.3 | 522.51 | 2680.6 | 708.66 | 2681.2 | 933.51 |

Table S7. Cont.

|  | J22 | | SZ-47 | | SZ-73 | | SZ-98 | | SZ-117 | | SZ-138 | |
| --- | --- | --- | --- | --- | --- | --- | --- | --- | --- | --- | --- | --- |
| No | m/z | Intens. | m/z | Intens. | m/z | Intens. | m/z | Intens. | m/z | Intens. | m/z | Intens. |
| 41 | 5460.4 | 709.33 | 10389.1 | 599.34 | 5461.2 | 534.1 | 3154.9 | 516.83 | 10389.5 | 701.31 | 5187.9 | 924.11 |
| 42 | 10137.4 | 695.68 | 5058.4 | 593.51 | 3154.4 | 530.36 | 3453.4 | 508.11 | 2225.1 | 678.77 | 2284.9 | 897.66 |
| 43 | 5247.9 | 694.72 | 3663.8 | 584.66 | 5060.9 | 522.64 | 2973.5 | 505.14 | 5459.5 | 672.14 | 3989 | 895 |
| 44 | 2976.8 | 673.99 | 5541.8 | 537.8 | 3468.9 | 505.42 | 2754.7 | 470 | 5315.5 | 667.91 | 3453.3 | 872.23 |
| 45 | 3986 | 656.48 | 2328.5 | 519.54 | 2962.9 | 445.63 | 5320.4 | 459.94 | 2977.8 | 617.46 | 5061.9 | 827.57 |
| 46 | 10391.6 | 605.9 | 2375.8 | 484.2 | 10139.3 | 406.58 | 3467.6 | 452.26 | 3665.7 | 566.09 | 2226 | 779.49 |
| 47 | 5754.9 | 574.56 | 10505.8 | 459.14 | 10391.9 | 336.93 | 3640.7 | 420.14 | 5543.7 | 542.89 | 3155.8 | 777.31 |
| 48 | 5060.6 | 554.46 | 10934.8 | 442.91 | 10938.3 | 243.47 | 7339.8 | 343.54 | 3108.3 | 524.77 | 2977.3 | 752.03 |
| 49 | 7056.4 | 407.36 | 7055.2 | 412.8 | 7487.3 | 223.18 | 7487.8 | 339.63 | 2754.5 | 465.74 | 2770.1 | 706.2 |
| 50 | 10511.4 | 367.93 | 5751 | 366.51 | 10509.3 | 219.57 | 11527 | 213.09 | 10506.7 | 427.8 | 3666.9 | 656.43 |

m/z - intensity values of top 50 major peaks were listed. It includes six isolates of M12 type (J22, SZ-47, SZ-73, SZ-98, SZ-117, SZ-138).
