# Supplementary material for: Identification and Cluster Analysis of Streptococcus pyogenes by MALDI-TOF Mass Spectrometry
Source: PLoS One. 2012 Nov 7;7(11):e47152. doi: 10.1371/journal.pone.0047152 (PMC3492366; doi:10.1371/journal.pone.0047152)
Supplement: Table S8 — Peaklist for M18 and M60 type isolates. m/z – intensity values of top 50 major peaks were listed. It includes four M18 type isolates (8617, 8629, 8634, 8643) and two M60 isolates (70206, E447). (DOCX) [file pone.0047152.s010.docx]

Table S8. Peaklist for M18 and M60 type isolates.

|  | 8617 | | 8629 | | 8634 | | 8643 | | 70206 | | E447 | |
| --- | --- | --- | --- | --- | --- | --- | --- | --- | --- | --- | --- | --- |
| No | m/z | Intens. | m/z | Intens. | m/z | Intens. | m/z | Intens. | m/z | Intens. | m/z | Intens. |
| 1 | 6834 | 30574.03 | 4562.2 | 22200.28 | 6832.7 | 16208.83 | 9528.4 | 11385.34 | 6476.3 | 28129.27 | 4451.9 | 1986.06 |
| 2 | 9529.4 | 16332.51 | 4452.4 | 17940.08 | 6898.3 | 11981.83 | 6313 | 5574.57 | 4563.1 | 23014.52 | 5396.9 | 1471.8 |
| 3 | 6927.5 | 6986.29 | 9531.8 | 17506.75 | 4560.5 | 11134.89 | 6736.7 | 5297.57 | 5222.5 | 8327.54 | 4560.6 | 1270.55 |
| 4 | 4758.6 | 6773.83 | 5363.5 | 16071.43 | 4451.1 | 8676.63 | 6926.5 | 5157.31 | 6514.4 | 7514.16 | 9528.9 | 1156.66 |
| 5 | 4561.4 | 6408.65 | 6738.4 | 10337.71 | 6736.9 | 7108.63 | 4451.3 | 4693.06 | 4601.5 | 6678.91 | 6313.4 | 1020.42 |
| 6 | 6313.5 | 5292.96 | 6802.3 | 10274.65 | 6800.2 | 6194.31 | 4560.5 | 4188.57 | 3236.8 | 3690.42 | 6802 | 949.02 |
| 7 | 6737.4 | 5278.08 | 4590.3 | 10135.09 | 5361.6 | 6097.83 | 4576.9 | 4172.06 | 6437.6 | 2525.02 | 6737.5 | 911.93 |
| 8 | 4451.6 | 4916.78 | 6314.5 | 9810.47 | 6938.2 | 5257.57 | 6799.3 | 3917.51 | 4290.4 | 2188.77 | 6842 | 849.06 |
| 9 | 3414.8 | 4780 | 6845.3 | 9519.01 | 9528.6 | 4983.86 | 5362.4 | 3599.26 | 5260.6 | 2040.31 | 5914.9 | 763.54 |
| 10 | 4577.2 | 4098.04 | 5958 | 8194.99 | 4587.9 | 4892.23 | 5377.6 | 3576.49 | 4449.9 | 1980.25 | 6897.4 | 522.66 |
| 11 | 5362.7 | 3894.63 | 8191 | 6078.34 | 4575.5 | 4229.37 | 8187.9 | 3166.8 | 4461.7 | 1837.1 | 7969.4 | 512.31 |
| 12 | 6219.3 | 2941.4 | 6900.4 | 5909.5 | 5956.3 | 4199.11 | 4758 | 3019.43 | 6831 | 1573.65 | 6942.8 | 486.64 |
| 13 | 8189.4 | 2878.61 | 7970.9 | 5871.61 | 6312 | 3694.09 | 6219.8 | 2692 | 2281.7 | 1248.11 | 5377.4 | 457.05 |
| 14 | 4536.5 | 2326.46 | 6947.4 | 5617.95 | 8187.1 | 2502.6 | 4465.9 | 2258.77 | 6897 | 1195.2 | 4757.3 | 429.71 |
| 15 | 5377.9 | 2300.95 | 7340.6 | 4770.05 | 3419.3 | 2210.91 | 5929.2 | 2116.03 | 4914.1 | 884.06 | 4593.8 | 421.66 |
| 16 | 5956.9 | 2200.87 | 5915.5 | 4224.6 | 5375.8 | 2162.69 | 3419.4 | 2041.57 | 6795.1 | 817.82 | 4512.9 | 417.8 |
| 17 | 4512.5 | 2195.42 | 4759.3 | 3321.22 | 5914.4 | 1906.89 | 4513.6 | 2012.49 | 2822 | 800.01 | 5955.9 | 399.25 |
| 18 | 5914.8 | 1692.96 | 9086.4 | 2848.96 | 3366.2 | 1706.69 | 4590.5 | 1897.77 | 6580.6 | 784.81 | 2698.7 | 381.07 |
| 19 | 7983.1 | 1646.38 | 6221.2 | 2778.47 | 4757.2 | 1690.06 | 5956.1 | 1786.71 | 2611.9 | 777.27 | 8188 | 374.27 |
| 20 | 3366.5 | 1505.44 | 9039.2 | 2642.55 | 5318.2 | 1421.71 | 7984.5 | 1771.86 | 5955.6 | 621.38 | 5353 | 334.07 |
| 21 | 4090.5 | 1502.95 | 3420.8 | 2059.01 | 4090.2 | 1080.51 | 4482.8 | 1762.2 | 2145.6 | 613.99 | 3418.4 | 306.36 |
| 22 | 5929.3 | 1453.27 | 10139.1 | 1821.64 | 5514.1 | 1052.6 | 4534.4 | 1704.14 | 2225 | 536.07 | 7336.6 | 298.45 |
| 23 | 3463.3 | 1428.71 | 10391.4 | 1719.91 | 7969.4 | 1047.2 | 7396.2 | 1649.4 | 3903.9 | 526.84 | 2226.4 | 285.48 |
| 24 | 7396.2 | 1283.72 | 2681.4 | 1646.94 | 2679.4 | 977.63 | 5913.6 | 1388.46 | 6311.3 | 525.44 | 4091.5 | 253.49 |
| 25 | 9083.7 | 1259.05 | 3367.7 | 1608.39 | 6363.5 | 963.26 | 9083.5 | 1384.97 | 6347.4 | 450.04 | 2280.4 | 241.93 |
| 26 | 2680.8 | 1073.41 | 3399.3 | 1480.86 | 2280.1 | 959.94 | 5973.2 | 1280.54 | 3396.1 | 443.76 | 3363.2 | 228.74 |
| 27 | 5186.7 | 1061.94 | 4091.6 | 1478.02 | 2226.1 | 879.23 | 4090.3 | 1195.77 | 6642.5 | 443.75 | 3980.9 | 218.73 |
| 28 | 3988.2 | 1052.94 | 5187.9 | 1417.66 | 5457.7 | 796.26 | 9039.6 | 1156.77 | 2745.4 | 428.79 | 2955 | 218.17 |
| 29 | 3154 | 1026.18 | 5467.4 | 1315 | 2976.5 | 765.09 | 3365.8 | 1156.51 | 2432.5 | 419.77 | 9084.5 | 217.59 |
| 30 | 5513.2 | 991.56 | 10512.3 | 1288.77 | 7985.5 | 729.11 | 5185.7 | 870.31 | 3446.6 | 396.03 | 5456.7 | 207.74 |
| 31 | 5059.8 | 987.48 | 10953.1 | 1245.94 | 3153.2 | 625.63 | 5513.9 | 865.37 | 2860.1 | 387.04 | 6350.4 | 207.48 |
| 32 | 9035.4 | 965.69 | 3982.2 | 1201.99 | 9035.1 | 623.89 | 5317.4 | 854.71 | 2977 | 371.23 | 9037.7 | 206.19 |
| 33 | 5320.1 | 957.59 | 3667.2 | 1191.92 | 6234.2 | 597.63 | 3154.6 | 835.94 | 5890.3 | 338.29 | 5186.9 | 206.08 |
| 34 | 3171.6 | 890.31 | 2226.5 | 1191.14 | 7337.5 | 577.14 | 3988.6 | 797.2 | 2926.1 | 336.81 | 3665.8 | 200.64 |
| 35 | 5460.4 | 850.9 | 2280.1 | 1175.33 | 9081.2 | 520.86 | 10136.1 | 762.49 | 2471.6 | 333.77 | 5060.2 | 178.55 |
| 36 | 2279.8 | 814.17 | 3470.6 | 1053.68 | 3665.6 | 512.66 | 5059.6 | 733 | 3677.6 | 306.5 | 5515.7 | 175.26 |
| 37 | 2976.3 | 797.01 | 2978.2 | 940.32 | 3982.1 | 501.51 | 3695.9 | 721.34 | 4088.4 | 302.91 | 3756.1 | 157.51 |
| 38 | 10134.1 | 776.24 | 3155.8 | 911.07 | 5184.6 | 476.83 | 2681.2 | 716.37 | 6939.9 | 292.64 | 6219.4 | 156.25 |
| 39 | 5245.7 | 733.98 | 3642.1 | 906.5 | 5059.5 | 413.51 | 5246.6 | 700.91 | 5910.2 | 287.65 | 10389.5 | 128.02 |
| 40 | 2225.4 | 731.15 | 2532.3 | 697.73 | 10136.6 | 295.71 | 10390.9 | 670.63 | 2496.5 | 287.48 | 10137.1 | 114.36 |

Table S8. Cont.

|  | 8617 | | 8629 | | 8634 | | 8643 | | 70206 | | E447 | |
| --- | --- | --- | --- | --- | --- | --- | --- | --- | --- | --- | --- | --- |
| No | m/z | Intens. | m/z | Intens. | m/z | Intens. | m/z | Intens. | m/z | Intens. | m/z | Intens. |
| 41 | 10390.6 | 694.01 | 2376.9 | 644.73 | 10935.8 | 247 | 6366.4 | 661.26 | 4331 | 287.24 | 8068.7 | 108.05 |
| 42 | 3694.6 | 647.32 | 9871.4 | 583.36 | 10389.3 | 236.29 | 5458.9 | 628.17 | 5512.1 | 184.66 | 6151.5 | 103.93 |
| 43 | 4024.5 | 627.82 | 7482.4 | 544.82 | 7199.3 | 210.51 | 2226.4 | 553.86 | 6734.5 | 158.99 | 9631.7 | 102.09 |
| 44 | 2758 | 627.37 | 11507.6 | 478.1 | 7471.2 | 179.2 | 2275.9 | 546.29 | 6216.1 | 154.99 | 10099.3 | 101.19 |
| 45 | 3108.6 | 605.07 | 12334 | 269.95 | 10503.9 | 145.77 | 2976.1 | 526.71 | 8185 | 135.65 | 7477.1 | 95.78 |
| 46 | 8828.1 | 489.05 | 12159.4 | 266.65 | 8830.3 | 124.31 | 10508.9 | 377.43 | 9526.2 | 110.62 | 10934.3 | 74.06 |
| 47 | 10937.9 | 435.03 | 13286.6 | 169.08 | 11504.1 | 92.29 | 10935.8 | 376.03 | 7964.9 | 93.31 | 10507.8 | 64.17 |
| 48 | 5756 | 383.61 | 13333.1 | 156.99 | 12151.2 | 84.86 | 7488.3 | 333.37 | 9666.9 | 89.13 | 7633.3 | 58.37 |
| 49 | 10507.1 | 347.43 | 15009.3 | 121.19 | 9858.4 | 83.46 | 7196.3 | 314.2 | 8985.8 | 75.68 | 12328.8 | 32.24 |
| 50 | 11524.9 | 192.74 | 14158.3 | 117.24 | 14422.9 | 31.94 | 5747.4 | 310.03 | 9810.2 | 39.75 | 11509.2 | 31.97 |

m/z - intensity values of top 50 major peaks were listed. It includes four M18 type isolates (8617, 8629, 8634, 8643) and two M60 isolates (70206, E447).
