# Supplementary material for: Identification and Cluster Analysis of Streptococcus pyogenes by MALDI-TOF Mass Spectrometry
Source: PLoS One. 2012 Nov 7;7(11):e47152. doi: 10.1371/journal.pone.0047152 (PMC3492366; doi:10.1371/journal.pone.0047152)
Supplement: Table S9 — Peaklist for M28 and M80 type isolates. m/z – intensity values of top 50 major peaks were listed. It includes three M28 type isolates (8619, 8624, 8625) and three M80 type isolates (8607, 8608, 8640). (DOCX) [file pone.0047152.s011.docx]

Table S9. Peaklist for M28 and M80 type isolates.

|  | 8619 | | 8624 | | 8625 | | 8607 | | 8608 | | 8640 | |
| --- | --- | --- | --- | --- | --- | --- | --- | --- | --- | --- | --- | --- |
| No | m/z | Intens. | m/z | Intens. | m/z | Intens. | m/z | Intens. | m/z | Intens. | m/z | Intens. |
| 1 | 4561.3 | 18656.7 | 9532.3 | 22327.16 | 9531.4 | 16217.1 | 4561.8 | 14935.18 | 6819.2 | 20353.77 | 9529.8 | 19644.6 |
| 2 | 9529.9 | 17496.83 | 4562.4 | 21356.57 | 4578.2 | 12653.76 | 4452.3 | 10769.52 | 4452.1 | 20268.89 | 4561.5 | 19421.15 |
| 3 | 6834.4 | 16511.24 | 6836 | 15517.73 | 6835.7 | 12141.08 | 6820 | 9170.51 | 6737.8 | 19161.13 | 6819.6 | 16465.12 |
| 4 | 4577.1 | 13517.28 | 4578.3 | 13997.12 | 4562.6 | 11524.21 | 9530.6 | 8997.44 | 4561.6 | 16984 | 6736.9 | 13229.47 |
| 5 | 4589.5 | 12037.64 | 6739.1 | 11459.33 | 4591.2 | 9102.07 | 5363.3 | 8911.45 | 6801.8 | 15682.76 | 4451.6 | 13129.42 |
| 6 | 6737.8 | 11415.73 | 6901.1 | 10383.38 | 6738.9 | 8616.35 | 4590.4 | 8688.13 | 5363 | 15390.87 | 6844.6 | 11428.54 |
| 7 | 4451.8 | 10557.78 | 4590.6 | 10070.82 | 6314.6 | 8122.54 | 4577.6 | 8299.1 | 6844.1 | 14491.4 | 4577 | 11176.06 |
| 8 | 6899.1 | 9654.89 | 4452.8 | 9709.14 | 6900.5 | 7463.92 | 6737.5 | 8051.3 | 6899.5 | 12370.14 | 4589.9 | 10931.13 |
| 9 | 6312.9 | 8648.97 | 6314.9 | 9620.65 | 4605.6 | 6893.57 | 6844.5 | 7873.62 | 5957.3 | 7853.89 | 6899.4 | 10492.95 |
| 10 | 4604.6 | 8460.61 | 8191 | 7152.74 | 4452.7 | 6716.59 | 6800.9 | 6578.96 | 4577.7 | 7302.8 | 6801.5 | 9700.56 |
| 11 | 5363.1 | 8426.2 | 5363.6 | 7048.45 | 5380 | 6506.55 | 6326.1 | 5099.96 | 5915.2 | 6994.92 | 8189.5 | 8229.19 |
| 12 | 6801.5 | 7923.28 | 5958.8 | 5844.4 | 5364.1 | 5856.8 | 6898.7 | 4721.9 | 4590.4 | 6528.01 | 5362.9 | 7958.77 |
| 13 | 5378.5 | 6822.44 | 4759.7 | 5762.83 | 8191 | 5381.24 | 7952.3 | 4668.31 | 6943.3 | 6052.01 | 7951.8 | 7843.54 |
| 14 | 8189.5 | 6726.34 | 5379.9 | 5350.83 | 4759.5 | 4849.44 | 8190.6 | 4539.2 | 8190 | 5881.05 | 6325 | 7146.23 |
| 15 | 5957.3 | 6212.99 | 4515.7 | 5228.11 | 4514.3 | 4148.85 | 5957.1 | 4162.82 | 9529 | 4754.59 | 4603.6 | 7066.95 |
| 16 | 4468.3 | 4750.7 | 6126 | 4350.18 | 5957.9 | 3689.16 | 6947.5 | 3205.2 | 5318.7 | 4126.28 | 5957.2 | 6629.77 |
| 17 | 4514.6 | 4452.38 | 6944.4 | 3969.03 | 7987.1 | 3382.18 | 7339.7 | 3116.9 | 7339.8 | 3312.15 | 6945.9 | 5481.71 |
| 18 | 7985.3 | 4239.08 | 7986.8 | 3942.28 | 5973.1 | 3356.23 | 5916.1 | 3037.98 | 7951.5 | 3281.72 | 4757.9 | 4810.56 |
| 19 | 5972.5 | 4118.86 | 5974.2 | 3911.65 | 7340.8 | 3334.16 | 4513.1 | 2624.42 | 6325.9 | 2992.69 | 5378.7 | 4432.56 |
| 20 | 6943.3 | 4103.99 | 7340.9 | 3429.59 | 6124.9 | 2874.64 | 6352.1 | 2301.75 | 4514.5 | 2945.03 | 5914.7 | 4079.56 |
| 21 | 4758.6 | 3983.08 | 9086.3 | 3327.43 | 3421.3 | 2524.25 | 5319.9 | 2255.22 | 3367.1 | 2629 | 9084.3 | 3528.78 |
| 22 | 6124.7 | 3686.23 | 9041.6 | 3233.26 | 5931.8 | 2428.6 | 6219.4 | 2018.96 | 5515.2 | 2517.75 | 5972.4 | 3526.9 |
| 23 | 7337.2 | 3413.8 | 5931.5 | 2699.19 | 9085.6 | 2278.38 | 9085.1 | 1700.84 | 3419.3 | 2413.68 | 6219.4 | 3338.32 |
| 24 | 5930.5 | 3067.72 | 3420.5 | 2467.61 | 9041.4 | 2088.48 | 4759.6 | 1531.18 | 3398.9 | 1967.42 | 5931.5 | 3208.82 |
| 25 | 3420.2 | 2941.82 | 4092 | 2183.22 | 3367.2 | 1819.32 | 3420.1 | 1505.48 | 5461.2 | 1747.79 | 4517.4 | 3082.54 |
| 26 | 9039 | 2881.77 | 10394.4 | 1857.21 | 5913.8 | 1737.12 | 8995.5 | 1279.03 | 8996 | 1675.61 | 6351.2 | 2600.01 |
| 27 | 9085.8 | 2806.84 | 10139.8 | 1845.76 | 4091.5 | 1602.68 | 3367.9 | 1181.74 | 2680.1 | 1651.11 | 8997 | 2521.47 |
| 28 | 5912.5 | 2641.9 | 3367 | 1731.79 | 10139.6 | 1364.25 | 4091.5 | 1167.29 | 3448.9 | 1550.7 | 10390.2 | 2429.75 |
| 29 | 5316.9 | 2202.15 | 5317.7 | 1639.66 | 10391 | 1194.1 | 3667.3 | 1006.23 | 4090.8 | 1517 | 3420.4 | 2317.84 |
| 30 | 6140.4 | 2095.03 | 5188 | 1575.39 | 3667.2 | 1193.93 | 10139.1 | 980.31 | 3666.7 | 1375.79 | 10136.1 | 2169.38 |
| 31 | 10137 | 1877.69 | 5516.8 | 1411.82 | 5187.5 | 1183.22 | 5516.7 | 936.68 | 4758.2 | 1229.46 | 3365.9 | 1984.66 |
| 32 | 4091.3 | 1846.77 | 5460.5 | 1405.6 | 3447.5 | 1174.69 | 9043.1 | 929.94 | 2226 | 1164.05 | 7338.4 | 1982.3 |
| 33 | 3366.6 | 1838.16 | 10940.8 | 1357.13 | 2688.3 | 1095.43 | 2681.4 | 895.97 | 6219.8 | 1024.46 | 4090.3 | 1972.16 |
| 34 | 10391.2 | 1833.79 | 2281.8 | 1282.58 | 3989.6 | 1093.56 | 10390.3 | 870.29 | 2977.4 | 981.01 | 9039.4 | 1863.54 |
| 35 | 5460.1 | 1389.83 | 5248 | 1246.18 | 2289.8 | 1074.63 | 5459.9 | 852.37 | 2274.6 | 972.64 | 5516.6 | 1844.96 |
| 36 | 5514.8 | 1366.96 | 10511.8 | 1235.24 | 3156.1 | 1024.7 | 5187.4 | 825.89 | 2182.3 | 961.65 | 10938 | 1705 |
| 37 | 5186 | 1352.71 | 3447.7 | 1128.74 | 5461.1 | 999.73 | 3972.1 | 766.37 | 2976.8 | 945.46 | 6366.7 | 1607.95 |
| 38 | 2280.4 | 1237.11 | 3991.1 | 1113.32 | 5516.7 | 951.99 | 10511 | 694.35 | 9082.9 | 941.69 | 5320.5 | 1485.31 |
| 39 | 2681.5 | 1180.72 | 5061.6 | 1059.24 | 5061.3 | 919.93 | 2980.8 | 606.89 | 10938.5 | 881.9 | 3971.7 | 1475.1 |
| 40 | 10938.7 | 1173.33 | 3155.3 | 1055.63 | 10510.6 | 752.28 | 5059.1 | 573.32 | 2483 | 865.34 | 10509.6 | 1427.47 |

Table S9. Cont.

|  | 8619 | | 8624 | | 8625 | | 8607 | | 8608 | | 8640 | |
| --- | --- | --- | --- | --- | --- | --- | --- | --- | --- | --- | --- | --- |
| No | m/z | Intens. | m/z | Intens. | m/z | Intens. | m/z | Intens. | m/z | Intens. | m/z | Intens. |
| 41 | 2689.5 | 1119.99 | 3668.3 | 973.53 | 10940.4 | 748.82 | 10938.9 | 543.34 | 2890.1 | 818.66 | 5186.9 | 1416.51 |
| 42 | 3448.8 | 1108.15 | 2690.3 | 972.73 | 2226.7 | 706.96 | 5742.1 | 478.35 | 9038.4 | 804.89 | 5460.3 | 1318.03 |
| 43 | 3666.5 | 1107.08 | 2978.7 | 865.11 | 4026.2 | 674.05 | 9634.9 | 405.33 | 2377.9 | 764.67 | 2280.3 | 1171.56 |
| 44 | 2289.5 | 1076.46 | 7493 | 753.01 | 2977.8 | 653.56 | 7486.2 | 291.64 | 4664.6 | 701.9 | 5061.1 | 1164.84 |
| 45 | 10509.5 | 1072.61 | 2755.4 | 670.45 | 11524.7 | 228.02 | 9870.6 | 239.56 | 2756.3 | 688.97 | 5245.7 | 1087.66 |
| 46 | 8065.4 | 1058.26 | 3058.9 | 580.39 | 12347.2 | 169.62 | 11510.8 | 189.18 | 3973.1 | 687.4 | 2226.2 | 1045.2 |
| 47 | 5061.5 | 1046.69 | 5751.7 | 577.45 | 13336.9 | 135.5 | 12336.4 | 126.38 | 2533 | 680.31 | 2681.1 | 986.94 |
| 48 | 3155.3 | 1038.03 | 11528.9 | 452.96 | 12125.7 | 119.79 | 13278.5 | 98.87 | 5755.2 | 624.48 | 2977.2 | 874.37 |
| 49 | 3987.6 | 999.22 | 12348.3 | 264.73 | 13280.4 | 107.26 | 13339.1 | 90.88 | 10139.1 | 512.47 | 7199.2 | 800.01 |
| 50 | 2226.7 | 895.36 | 12125.1 | 262.94 | 13552.2 | 89.44 | 14152.4 | 68.68 | 10390.4 | 352.96 | 5743.6 | 795.65 |

m/z - intensity values of top 50 major peaks were listed. It includes three M28 type isolates (8619, 8624, 8625) and three M80 type isolates(8607, 8608, 8640).
