# Supplementary material for: Identification and Cluster Analysis of Streptococcus pyogenes by MALDI-TOF Mass Spectrometry
Source: PLoS One. 2012 Nov 7;7(11):e47152. doi: 10.1371/journal.pone.0047152 (PMC3492366; doi:10.1371/journal.pone.0047152)
Supplement: Table S10 — Peaklist for M77, M101, M6 and M58 type isolates. m/z – intensity values of top 50 major peaks were listed. It includes two M77 type isolates (8630, 8638), two M101 type isolates (8633, 8641), one M6 type isolate (8637) and one M58 type isolate (G519). (DOCX) [file pone.0047152.s012.docx]

Table S10. Peaklist for M77, M101, M6 and M58 type isolates.

|  | 8630 | | 8638 | | 8633 | | 8641 | | 8637 | | G519 | |
| --- | --- | --- | --- | --- | --- | --- | --- | --- | --- | --- | --- | --- |
| No | m/z | Intens. | m/z | Intens. | m/z | Intens. | m/z | Intens. | m/z | Intens. | m/z | Intens. |
| 1 | 4560.2 | 15063.57 | 4560.4 | 10421.34 | 4559.7 | 8063.2 | 9528 | 14191.8 | 6833.3 | 23181.77 | 9528.9 | 12549.17 |
| 2 | 9526.9 | 12728.09 | 9527.7 | 8916.91 | 9526.3 | 7903.31 | 4560.6 | 9946.26 | 4576.5 | 9156.86 | 4560.6 | 12374.09 |
| 3 | 4450.7 | 12464.1 | 4450.8 | 8373.34 | 4450.6 | 5938.71 | 6832 | 8401.57 | 9528.4 | 8265.69 | 4450.9 | 11955.29 |
| 4 | 4588.4 | 8176.12 | 6830.4 | 6575.34 | 5361.5 | 5216.91 | 6842.6 | 7168.29 | 6736.6 | 7485.34 | 5362 | 8049.37 |
| 5 | 6830.7 | 8123.7 | 5361.9 | 6056.63 | 6311.6 | 4906.29 | 4451 | 7025.83 | 6312.2 | 6764.37 | 6833.4 | 7811.4 |
| 6 | 6311.7 | 7107.33 | 6842.2 | 5822.4 | 6842.3 | 4899.06 | 6312.3 | 6550.14 | 6898.1 | 6320.89 | 6736.8 | 7591.14 |
| 7 | 6735.6 | 6938.74 | 6312 | 5646.66 | 6831.8 | 4848.83 | 6735.7 | 6067.43 | 4560.6 | 6281.23 | 6800.2 | 6811.6 |
| 8 | 6799.7 | 6009.38 | 4587.7 | 5119.29 | 6735.5 | 4611.09 | 4757.7 | 5804.06 | 6801.1 | 5319.6 | 6842.5 | 6661.86 |
| 9 | 5925.7 | 5886.59 | 6736.5 | 5074.74 | 4587.5 | 4371.14 | 4576.4 | 5677.26 | 4590.4 | 4875.37 | 6312.5 | 6536.2 |
| 10 | 5361.3 | 5507.67 | 4575.5 | 4513.26 | 4575.3 | 4147.57 | 5362.2 | 5573.69 | 5377 | 4527.54 | 5956.4 | 6512.97 |
| 11 | 4757.2 | 4562.55 | 6799.8 | 4491.34 | 6799.6 | 3592.26 | 4588.6 | 4892.83 | 4604.4 | 4303.71 | 6898.5 | 4912.94 |
| 12 | 8203.3 | 3673.48 | 5925 | 3391.29 | 6896.3 | 2848.49 | 6897.1 | 4892.2 | 4450.5 | 3936.09 | 4588.7 | 4285.43 |
| 13 | 7967.7 | 3312.47 | 4757.2 | 2731.6 | 5956 | 2713.86 | 6799.6 | 4291.91 | 8187.6 | 3848.03 | 7968.7 | 3892.94 |
| 14 | 6943.7 | 3205.68 | 8203.9 | 2661.74 | 5376.4 | 2605.69 | 8187.5 | 3725.49 | 6219.1 | 3741.91 | 6944.6 | 3877.37 |
| 15 | 4511.6 | 2620.49 | 7336.5 | 2363.8 | 8186.9 | 2458 | 5377.8 | 3475.91 | 4513.5 | 3722.37 | 4758.2 | 3685.57 |
| 16 | 3419 | 2443.58 | 6944.1 | 2126.51 | 4757.5 | 2443.71 | 5955.8 | 3261.91 | 5971.1 | 3642.46 | 8188.5 | 3616 |
| 17 | 6218.4 | 1968.51 | 5376.9 | 1980.26 | 6944.7 | 2162.46 | 6218.5 | 2990.26 | 5362.4 | 3509.86 | 7337.5 | 2043.54 |
| 18 | 3365.7 | 1842.61 | 7968.4 | 1949.83 | 7336.9 | 2105.09 | 7337.9 | 2637 | 3414.2 | 3442.17 | 6349.8 | 1950.94 |
| 19 | 4098 | 1743.77 | 6218.5 | 1831.17 | 7983.4 | 1531.83 | 6944.7 | 2249.71 | 4757.5 | 3295.51 | 5912.9 | 1737.29 |
| 20 | 9080.7 | 1636.63 | 5939.7 | 1785.66 | 6218.4 | 1441.34 | 3419.6 | 2196.94 | 5956.5 | 3181.94 | 3419 | 1716.14 |
| 21 | 9036.1 | 1593.26 | 4511.5 | 1722.26 | 5911.6 | 1438.14 | 4514.3 | 2127.03 | 6366.3 | 2482.29 | 6219.1 | 1632.66 |
| 22 | 3397.1 | 1543.8 | 7983 | 1642 | 6349.5 | 1297.14 | 7983.6 | 2020.97 | 7983.6 | 2346 | 9083.4 | 1624.66 |
| 23 | 6349.8 | 1372.5 | 3419 | 1379.31 | 7966.9 | 1290.97 | 4089.8 | 1654.77 | 7336.8 | 1842.23 | 3366.1 | 1442.8 |
| 24 | 2280.1 | 1323.54 | 9037.9 | 1192.97 | 3419.5 | 1264.11 | 9083.1 | 1639.54 | 9082.6 | 1710.17 | 8996.2 | 1397.14 |
| 25 | 5200.1 | 1210.28 | 3366.7 | 1170.14 | 5314.6 | 1154.11 | 3366 | 1468.83 | 3366.6 | 1675.43 | 3398.7 | 1327.69 |
| 26 | 2224.5 | 1169.49 | 9080.9 | 1147.54 | 3365.8 | 1121.6 | 5971.1 | 1428.57 | 4089 | 1608.37 | 4089.3 | 1307.74 |
| 27 | 3468.4 | 1088.77 | 5317.5 | 1082.57 | 9081.1 | 1041.6 | 5930.3 | 1416.74 | 9038.6 | 1581.23 | 2680.6 | 1251.23 |
| 28 | 2961.7 | 1084.42 | 4096.5 | 1040.4 | 4089 | 995.94 | 6351.4 | 1300.34 | 5317.4 | 1407.09 | 5186.8 | 1208.91 |
| 29 | 5316.8 | 1067.91 | 2680.1 | 943.29 | 2680.2 | 901.11 | 5186 | 1287.6 | 5912.2 | 1326.94 | 3979.8 | 1159.06 |
| 30 | 5246.4 | 1005.54 | 5200.2 | 933.43 | 3666.3 | 865.97 | 5914.1 | 1255.74 | 5926.4 | 1209.46 | 2976.6 | 1095.17 |
| 31 | 3987.5 | 999.15 | 3664.4 | 874.17 | 5513.4 | 852.74 | 5515.2 | 1179.57 | 7998.7 | 1102.17 | 5458.7 | 1048.91 |
| 32 | 10414.4 | 971.44 | 2225.6 | 855.4 | 2279.8 | 843.31 | 7968.8 | 1170.89 | 3155.5 | 1056.8 | 10134.9 | 1040.66 |
| 33 | 5059.7 | 960.61 | 6349.1 | 836 | 5186.4 | 834.34 | 5060 | 1136.6 | 5185.5 | 1048.43 | 2225.8 | 999.71 |
| 34 | 2679.8 | 957.96 | 2280.4 | 772.63 | 8993 | 810 | 10134.5 | 1128.03 | 5245.8 | 1029.09 | 10390.3 | 888.83 |
| 35 | 3978.8 | 943.93 | 5244.7 | 749.91 | 2226.4 | 698.37 | 2680.9 | 1118.63 | 3987.7 | 1008.71 | 10936.7 | 813.26 |
| 36 | 10133.7 | 921.99 | 10134 | 736.71 | 3155.6 | 690.77 | 5321.1 | 1089.89 | 5458.5 | 918.31 | 8065.7 | 783.2 |
| 37 | 3155 | 866.59 | 5458.2 | 718.74 | 10132.9 | 688.86 | 5245.4 | 1050.14 | 2280.2 | 891.43 | 5244.3 | 770.46 |
| 38 | 5458.3 | 854.18 | 10414.9 | 704.11 | 10387.1 | 667.43 | 3155.1 | 1041.74 | 3667.1 | 875 | 2280.8 | 748.46 |
| 39 | 5512 | 831.07 | 3154.6 | 689.54 | 3979.6 | 631.37 | 2280.2 | 1041 | 5530.6 | 858.14 | 5058.5 | 740.46 |
| 40 | 2294.2 | 815.67 | 5060.4 | 668.97 | 5457.9 | 596.86 | 10388.5 | 1030.63 | 5059.2 | 837.09 | 3155.6 | 738.4 |

Table S10. Cont.

|  | 8630 | | 8638 | | 8633 | | 8641 | | 8637 | | G519 | |
| --- | --- | --- | --- | --- | --- | --- | --- | --- | --- | --- | --- | --- |
| No | m/z | Intens. | m/z | Intens. | m/z | Intens. | m/z | Intens. | m/z | Intens. | m/z | Intens. |
| 41 | 10506.7 | 646.48 | 3979.3 | 658.8 | 5058.8 | 589.54 | 3666.7 | 1027 | 2984.2 | 823 | 3665.1 | 708.37 |
| 42 | 4662.1 | 601.26 | 2960.9 | 616.49 | 2975.2 | 570.6 | 8995.1 | 1020.77 | 2754.5 | 812.34 | 10102.7 | 648.4 |
| 43 | 7335.9 | 518.74 | 5514.5 | 607.71 | 10500.2 | 394.43 | 5460.2 | 946.4 | 5515.1 | 796.91 | 10506.2 | 597.26 |
| 44 | 10934.7 | 503.89 | 7197.7 | 484.23 | 8827.5 | 359.66 | 3987.5 | 934.66 | 10134.8 | 765.54 | 7053.7 | 577.2 |
| 45 | 5740.5 | 495.94 | 10507.6 | 473.66 | 10931.3 | 324.09 | 9038.9 | 898.77 | 2681.3 | 732.8 | 4664.3 | 576.09 |
| 46 | 7196.4 | 473.51 | 10935.6 | 466.34 | 7195.8 | 280.31 | 2976.7 | 864.69 | 2225.6 | 607.69 | 2755 | 500.06 |
| 47 | 7479.2 | 463.96 | 2755.9 | 452.91 | 11503.9 | 211.4 | 8828.6 | 769.63 | 10387.1 | 553.6 | 8829.3 | 498.51 |
| 48 | 3753.4 | 406.25 | 8827.7 | 372.51 | 7479.8 | 208.2 | 5750 | 752.34 | 10509.4 | 483.63 | 7481.9 | 454.43 |
| 49 | 9862.8 | 230.72 | 7483.4 | 292.03 | 9864.9 | 182.83 | 10935.5 | 724.49 | 7487.5 | 469.89 | 5744.1 | 448.46 |
| 50 | 11504.9 | 188.59 | 9863.7 | 241.14 | 12148.1 | 143.2 | 2226.1 | 721.29 | 10934.3 | 430 | 7197.7 | 375.94 |

m/z - intensity values of top 50 major peaks were listed. It includes two M77 type isolates (8630, 8638), two M101 type isolates (8633, 8641), one M6 type isolate (8637) and one M58 type isolate (G519).
