# Supplementary material for: Identification and Cluster Analysis of Streptococcus pyogenes by MALDI-TOF Mass Spectrometry
Source: PLoS One. 2012 Nov 7;7(11):e47152. doi: 10.1371/journal.pone.0047152 (PMC3492366; doi:10.1371/journal.pone.0047152)
Supplement: Table S11 — Peaklist for M63, M64, M75, M86 and M95 type isolates. m/z – intensity values of top 50 major peaks were listed. It includes one M63 type isolates (G302), one M64 type isolates (8626), one M75 type isolates (8627), one M86 type isolates (8642), one M95 type isolates (C39). (DOCX) [file pone.0047152.s013.docx]

Table S11. Peaklist for M63, M64, M75, M86 and M95 type isolates.

|  | G302 | | 8626 | | 8627 | | 8642 | | C39 | |
| --- | --- | --- | --- | --- | --- | --- | --- | --- | --- | --- |
| No | m/z | Intens. | m/z | Intens. | m/z | Intens. | m/z | Intens. | m/z | Intens. |
| 1 | 9526.8 | 10524.89 | 5364.3 | 19628.97 | 4561.9 | 11672.67 | 4562 | 10346.26 | 4559.7 | 16478.8 |
| 2 | 4449.2 | 8572.9 | 4452.9 | 17697.84 | 9531.7 | 9910.46 | 9530.1 | 9331.63 | 4449.9 | 9352.6 |
| 3 | 5361.4 | 8118.47 | 9532.3 | 15443.86 | 6900.6 | 8337.25 | 6834.7 | 7125 | 9524.8 | 6324 |
| 4 | 6832.2 | 5434.23 | 4562.5 | 14368.51 | 4577.6 | 7855.12 | 4452.3 | 6276.91 | 5360.5 | 6121.09 |
| 5 | 4560 | 5082.98 | 6739.5 | 11531.55 | 4452.5 | 7480.3 | 4589.6 | 6264.89 | 6797.4 | 4409.86 |
| 6 | 6841.6 | 4689.44 | 6802.9 | 10916.14 | 4590 | 7164.15 | 6313.9 | 5120.8 | 4587.6 | 4355.03 |
| 7 | 6736.1 | 4210.5 | 6846.2 | 10496.37 | 6738.7 | 6486.34 | 6738 | 4978.71 | 6734.5 | 4083.11 |
| 8 | 6312 | 3992.3 | 6315.1 | 10107.76 | 6845.3 | 6217.32 | 6899 | 4549.6 | 5954.4 | 3581.54 |
| 9 | 6798.1 | 3290.86 | 7971.7 | 8867.39 | 6314.4 | 5902.23 | 5363.5 | 4447.71 | 6830.9 | 3542.63 |
| 10 | 5955.3 | 3268.88 | 7341 | 6860.59 | 5363.5 | 5487.23 | 4577.7 | 4340.49 | 6322.6 | 3497.11 |
| 11 | 7967.3 | 2577.4 | 5958.7 | 6510.06 | 4759.1 | 4300.19 | 6802.1 | 3748.54 | 6840.7 | 3428.29 |
| 12 | 6943.4 | 2480.03 | 5915.9 | 6466.71 | 6801.4 | 4210.82 | 4759.5 | 3109.91 | 6895.8 | 3261.51 |
| 13 | 4757.2 | 2396.54 | 4589.9 | 6380.52 | 6818.7 | 4158.92 | 5957.2 | 3063.23 | 7966.3 | 2977.34 |
| 14 | 8187.3 | 2279.29 | 8191.8 | 5913.44 | 5958.3 | 4132.57 | 8188.8 | 2598.63 | 6942.7 | 2911.37 |
| 15 | 6895.4 | 2108.66 | 6947.6 | 5033.67 | 8191.1 | 3760.84 | 5378.1 | 2294.23 | 8185.4 | 2369.49 |
| 16 | 7337.2 | 1945.44 | 4759.8 | 4435.1 | 5379.3 | 3664.75 | 6944.7 | 2166.94 | 4756.4 | 2293.06 |
| 17 | 4587.9 | 1649.87 | 5322.9 | 3494.07 | 6221.2 | 2267.77 | 4515.5 | 1689.4 | 5911.5 | 2090.14 |
| 18 | 5913.3 | 1298.36 | 6900.7 | 3402.27 | 7987.3 | 2078.66 | 6220.6 | 1614.34 | 6217.1 | 1904.14 |
| 19 | 3418.8 | 1075.37 | 9085.3 | 3008.05 | 3420.8 | 1893.25 | 3420.8 | 1518.29 | 6348.4 | 1197.14 |
| 20 | 6218.2 | 1072.03 | 9041.7 | 2847.47 | 3367 | 1891.59 | 7339.3 | 1388.6 | 3417.4 | 1194.69 |
| 21 | 2679.2 | 1042.43 | 3421.5 | 2686.25 | 4090.7 | 1873.96 | 6350.8 | 1377.66 | 2679.7 | 1139.46 |
| 22 | 9081.6 | 886.91 | 2681.9 | 2548.1 | 3447.9 | 1594.09 | 7986.4 | 1372.89 | 3979.3 | 1134.71 |
| 23 | 9036.6 | 874.15 | 10393.7 | 2543.63 | 7340.3 | 1379.26 | 4091.9 | 1205.91 | 2279.8 | 1122.91 |
| 24 | 4090.2 | 829.51 | 10139.3 | 2253 | 5917.2 | 1302.24 | 3366.7 | 1188.71 | 4088.6 | 1108.11 |
| 25 | 3365.6 | 824.28 | 3367.1 | 2140.26 | 9085.9 | 1296.91 | 5972 | 1176.26 | 2225.3 | 987.49 |
| 26 | 5464.3 | 774.85 | 3668 | 2129.64 | 5188.3 | 1095.21 | 2977.8 | 1162.69 | 5319.5 | 915.83 |
| 27 | 5198.9 | 713.57 | 5188.2 | 2084.22 | 9055.9 | 1082.52 | 5913.6 | 1153.89 | 9079.4 | 914.4 |
| 28 | 3980.8 | 683.84 | 3399 | 2043.7 | 3398.9 | 1073.46 | 9084.9 | 1082.86 | 3364.3 | 912.74 |
| 29 | 5243.4 | 665.95 | 5460.9 | 2010.81 | 2680.8 | 1028.22 | 5188.5 | 969.43 | 3160.6 | 858.11 |
| 30 | 3665.4 | 646.29 | 3981.7 | 2002.11 | 5247.2 | 1021.17 | 7968.3 | 889.09 | 2977 | 850.89 |
| 31 | 10414.1 | 646.13 | 5248 | 1908.9 | 3988.3 | 991.55 | 2680.9 | 861.97 | 4660.9 | 803.66 |
| 32 | 10133.7 | 616.77 | 4091.9 | 1870.83 | 3155 | 984.24 | 5319.4 | 847.8 | 5184.3 | 708.03 |
| 33 | 3468.4 | 584.24 | 10941 | 1781.5 | 5068.8 | 973 | 3989.4 | 834.23 | 5244.2 | 646.11 |
| 34 | 2977.7 | 574.28 | 6221 | 1728.24 | 2281 | 951.27 | 2226.4 | 788 | 10143.4 | 611.34 |
| 35 | 2224.5 | 557.6 | 2226.7 | 1600.96 | 2977.6 | 881.08 | 5460.4 | 787.29 | 7335.7 | 597.43 |
| 36 | 5057.9 | 546.37 | 10511.5 | 1527.05 | 5459.2 | 865.78 | 2280.2 | 767.8 | 2954.2 | 551.46 |
| 37 | 7196.2 | 530.22 | 5061.8 | 1446.13 | 3666.3 | 840.82 | 3155.4 | 763.91 | 10385.8 | 529.03 |
| 38 | 8062.2 | 483.76 | 2978.8 | 1425.8 | 10154.1 | 740.63 | 5060 | 738.6 | 8991 | 519 |
| 39 | 2279.8 | 425.55 | 3156.1 | 1335.41 | 2225.4 | 707.08 | 10138 | 719.57 | 5065.1 | 509.17 |
| 40 | 10104.1 | 390.48 | 3471.8 | 1279.02 | 10394 | 685.37 | 5246.2 | 703.09 | 9035.5 | 503.17 |

Table S11. Cont.

|  | G302 | | 8626 | | 8627 | | 8642 | | C39 | |
| --- | --- | --- | --- | --- | --- | --- | --- | --- | --- | --- |
| No | m/z | Intens. | m/z | Intens. | m/z | Intens. | m/z | Intens. | m/z | Intens. |
| 41 | 3153.8 | 380.95 | 2533.1 | 1245.51 | 5513.9 | 607.92 | 8997.8 | 685.63 | 5457.7 | 502.03 |
| 42 | 5741.4 | 378.2 | 12158.7 | 1041.76 | 2755.2 | 556.83 | 3666.2 | 670.43 | 3664.8 | 424.97 |
| 43 | 8827.5 | 371.32 | 6154.4 | 1033.03 | 5753.7 | 488.89 | 10391.3 | 585.23 | 6148.6 | 416.49 |
| 44 | 7052.9 | 369.3 | 7201.6 | 1016.51 | 10511.3 | 422.1 | 9039.4 | 511.34 | 8067.8 | 405.63 |
| 45 | 10505.1 | 342.74 | 7484.9 | 976.07 | 9634 | 392.7 | 6159.1 | 450.6 | 10099.6 | 383.06 |
| 46 | 9868 | 298.15 | 9870.4 | 911.59 | 10939 | 366.32 | 5515.2 | 446.03 | 10503.9 | 344.57 |
| 47 | 7479.2 | 286.73 | 5749.7 | 868.41 | 7201.7 | 348.63 | 10109.6 | 418.83 | 7477.9 | 324.11 |
| 48 | 6151.2 | 271.7 | 11523.1 | 854.1 | 7486.1 | 308.66 | 5742.2 | 379.2 | 8824.8 | 322.8 |
| 49 | 10934.2 | 268.92 | 7056.7 | 817.74 | 10972.7 | 232.67 | 10937.1 | 358.66 | 5741.9 | 292.89 |
| 50 | 4664.2 | 264.5 | 12332.9 | 595.12 | 8830.7 | 200.47 | 7200.3 | 342.86 | 10932.2 | 257 |

m/z - intensity values of top 50 major peaks were listed. It includes one M63 type isolates (G302), one M64 type isolates (8626), one M75 type isolates (8627), one M86 type isolates (8642), one M95 type isolates (C39).
